# Dental size variation in admixed Latin Americans: Effects of age, sex and genomic ancestry

**DOI:** 10.1371/journal.pone.0285264

**Published:** 2023-05-04

**Authors:** Guangrui Yang, Yingjie Chen, Qing Li, Daniel Benítez, Luis Miguel Ramírez, Macarena Fuentes-Guajardo, Tsunehiko Hanihara, G. Richard Scott, Victor Acuña Alonzo, Rolando Gonzalez Jose, Maria Catira Bortolini, Giovanni Poletti, Carla Gallo, Francisco Rothhammer, Winston Rojas, Clément Zanolli, Kaustubh Adhikari, Andres Ruiz-Linares, Miguel Delgado

**Affiliations:** 1 Ministry of Education Key Laboratory of Contemporary Anthropology and Collaborative Innovation Center of Genetics and Development, School of Life Sciences and Human Phenome Institute, Fudan University, Shanghai, China; 2 Department of Anthropology, University of Kentucky, Lexington, Kentucky, United States of America; 3 Facultad de Odontología, Universidad de Antioquia, Medellín, Colombia; 4 Departamento de Tecnología Médica, Facultad de Ciencias de la Salud, Universidad de Tarapacá, Arica, Chile; 5 Department of Anatomy, Kitasato University School of Medicine, Sagamihara, Japan; 6 Department of Anthropology, University of Nevada Reno, Reno, Nevada, United States of America; 7 Molecular Genetics Laboratory, National School of Anthropology and History, Mexico City, Mexico; 8 Instituto Patagónico de Ciencias Sociales y Humanas, Centro Nacional Patagónico, CONICET, Puerto Madryn, Argentina; 9 Departamento de Genética, Universidade Federal do Rio Grande do Sul, Porto Alegre, Brasil; 10 Laboratorios de Investigación y Desarrollo, Facultad de Ciencias y Filosofía, Universidad Peruana Cayetano Heredia, Lima, Perú; 11 Instituto de Alta Investigación, Universidad de Tarapacá, Arica, Chile; 12 GENMOL (Genética Molecular), Universidad de Antioquia, Medellín, Colombia; 13 CNRS, MCC, PACEA, UMR, Pessac, France; 14 School of Mathematics and Statistics, Faculty of Science, Technology, Engineering and Mathematics, The Open University, Milton Keynes, United Kingdom; 15 Laboratory of Biocultural Anthropology, Law, Ethics, and Health (Centre National de la Recherche Scientifique and Etablissement Français du Sang, UMR-7268), Aix-Marseille University, Marseille, France; 16 División Antropología, Facultad de Ciencias Naturales y Museo, Universidad Nacional de La Plata, La Plata, República Argentina; 17 Consejo Nacional de Investigaciones Científicas y Técnicas, CONICET, Buenos Aires, República Argentina; The Cyprus Institute, CYPRUS

## Abstract

Dental size variation in modern humans has been assessed from regional to worldwide scales, especially under microevolutionary and forensic contexts. Despite this, populations of mixed continental ancestry such as contemporary Latin Americans remain unexplored. In the present study we investigated a large Latin American sample from Colombia (N = 804) and obtained buccolingual and mesiodistal diameters and three indices for maxillary and mandibular teeth (except third molars). We evaluated the correlation between 28 dental measurements (and three indices) with age, sex and genomic ancestry (estimated using genome-wide SNP data). In addition, we explored correlation patterns between dental measurements and the biological affinities, based on these measurements, between two Latin American samples (Colombians and Mexicans) and three putative parental populations: Central and South Native Americans, western Europeans and western Africans through PCA and DFA. Our results indicate that Latin Americans have high dental size diversity, overlapping the variation exhibited by the parental populations. Several dental dimensions and indices have significant correlations with sex and age. Western Europeans presented closer biological affinities with Colombians, and the European genomic ancestry exhibited the highest correlations with tooth size. Correlations between tooth measurements reveal distinct dental modules, as well as a higher integration of postcanine dentition. The effects on dental size of age, sex and genomic ancestry is of relevance for forensic, biohistorical and microevolutionary studies in Latin Americans.

## Introduction

As the most abundant and best-preserved remains in the archaeological and forensic record, teeth have been used extensively in anthropological studies. The investigation of metric and nonmetric traits provides important insights into the biological diversity and evolution of living and extinct hominins [1–8]. In this regard, because tooth measurements are well defined, replicable and amenable to multivariate analysis, and vary according to expected patterns of neutral genetic data [9, 10], dental metric diversity has been widely investigated among modern human populations from bioarchaeological, evolutionary and forensic perspectives [7, 11–13].

The reconstruction of local and regional population histories through the study of biological affinities and microevolutionary dynamics, as well as the estimation of age, sex and genetic ancestry, are major topics in current odontometric research [7, 13–16]. Most of these studies have focused on populations classified using both discrete (e.g., European-Americans and African-Americans) [16] and continental population categories (e.g., Africans, Europeans, Asians, etc.) [7, 11–13, 17]. However, few odontometric investigations have been performed in individuals of mixed continental ancestry, such as Latin Americans [18]. Accordingly, the dental size variability of contemporary Latin Americans, especially regarding its usefulness to differentiate people with diverse ancestries, sexes, and ages remains to be elucidated.

Since Latin America has a long history of extensive admixture, mainly between Native Americans, Europeans, and Africans, populations from this region exhibit high levels of genetic and phenotypic diversity. Accounting for this high diversity is challenging for many forensic and historical studies, including efforts of human identification in a region that has often suffered from widespread violence, resulting in many thousands of unidentified victims. The aim of this work is to evaluate the effect of sex, age and genomic ancestry on tooth size in a large Latin American population sample in order to provide a baseline for future forensic, biohistorical and microevolutionary studies.

## Materials and methods

### Subjects of study

We studied a sample of 804 individuals of both sexes (women/men = 446/358) aged 18–40 (mean = 22.7 years) recruited in Medellín, Colombia (hereafter Colombians). Medellín is one of the largest cities in Colombia located in the northeast of the country in the Department of Antioquia whose population is composed mainly of persons of mixed continental ancestry, mostly people of European and Native American ancestry plus a small African contribution. The foundation of Medellín dates back to 17th century and the population interactions involved European men and Native American women [19–21] During the colonial period, the Medellín population remained relatively isolated which led to internal growth although during recent times the region experienced immigration from distinct regional and extra-regional sources [20] Previous genetic studies showed admixture dynamics during colonial and postcolonial times characterized by asymmetric gene flow and little population substructure [19, 21–23].

Of these individuals, ~275 had been recruited previously as part of the CANDELA cohort (Consortium for the Analysis of the Diversity and Evolution of Latin America, http://www.ucl.ac.uk/silva/candela) and were recontacted for this new study [24]. Information on relevant covariates age, sex and height (a proxy of body size) was obtained during volunteer interview. This research was approved by the Bioethics committee of Universidad de Antioquia School of Odontology (Colombia). All participants provided written informed consent.

### Comparative dental data

A population reference dataset collected by T.H was included to evaluate the phenotypic similarity/dissimilarity between Latin Americans and their putative parental populations [12]. This dataset includes Native Americans (N = 368), Europeans (N = 332) and Africans (N = 205), which came from areas that contributed extensively to admixture in Latin America including Central and South America (Native Americans from Mexico, Caribbean islands, Colombia, Venezuela, Ecuador, South Central Andes and southwest US), Western Europe (England, France, Germany, Spain and Italy) and Western Africa (Cameroon, Congo, Gabon, Ghana, Guinea and Sierra Leone). One additional recent sample from Mexico (hereafter Mexicans) (N = 57) from the reference dataset was included in order to evaluate the degree of differentiation among Latin Americans and their association with the parental populations. The Mexican sample investigated came from Chihvahva, Yucatan, Jarasco, Cora, Yaqui, Sierra Mazagan, Sonora, Tayopa, Zacatenco and Valley of Mexico. Most of the samples integrating this reference dataset are from collections of known age and sex and in such cases where this information was not available, T.H. [12] used standard osteological techniques to infer these covariates [25, 26] We included individuals from samples younger than 2000 years to avoid temporal biases. Detailed information on the composition of the comparative dataset, such as country of origin, ethnic affiliation, and cultural background is given in Hanihara and Ishida [12].

### Data collection

Dental plaster casts were obtained at the QST lab, Facultad de Odontología, Universidad de Antioquia (Medellín, Colombia) by L.M.R following international protocols. Because the persons investigated are mostly young and middle aged adults (mean = 22.7 years), they presented slight to moderate tooth wear (stages 1–4). Here we used the modified method [27] of occlusal surface wear stages adapted to living human populations [28].

Dental plaster casts were obtained using high quality materials such as alginate (hydrogum fast setting elastic alginate—Zhermack) and dental stone (elite ortho white—Zhermack). The casts were arranged in pairs and stored in a dry environment. Subsequently, after drying for 7 days the dental plaster casts were scanned *in situ* to obtain high-resolution 3D dental models. Two different high precision scanners were used: 1) DAVID SLS-2 structured light 3Dscanner (DAVID Vision Systems, Koblenz, Germany) and 2) A blue light portable 3D Artec Space Spider scanner (Artec 3D, Santa Clara, California). For DAVID SLS-2 all scans were carried out at the highest resolution, reported by the manufacturer as 80-μm point distance using 14 photograms per model. Dedicated supplier software, David 3D scanner Pro v 4.5.3.1374, was used for data acquisition, scan merging and digital model generation using the software automatic option. For Artec Space Spider the scans were conducted at 0.1 mm 3D resolution and 0.05 mm 3D accuracy. The Artec Studio software v15 was used for data processing using the manufacturer recommendations and specifications. High resolution 3D dental models were then exported as stereo-lithography (STL) files including volume, color and texture. A sub-sample of 15 virtual and physical plaster casts for the maxillary dentition (right side) was selected randomly to verify measurement accuracy. Mesiodistal diameters of UI1, UI2, UC, UP3, and UP4 were selected for comparison. The measurements were obtained using a Mitutoyo digital caliper (0.01 mm) in dental plaster casts and through the Meshlab software v. 2021.05 [29] in virtual models. Both sets of measurements (in physical and virtual models) were compared using a paired *t*-test (for systematic error) and error percentages (for random error) following Yong et al [30].

### Tooth measurements

We obtained two crown diameters (in mm) maximum mesiodistal (MD) and maximum buccolingual (BL) in upper and lower permanent dentitions defined by Hillson [31] as the maximum distance between two parallel planes, tangential to the most mesial and most distal points of the crown side and the maximum distance between two parallel planes, one tangential to the lingual crown side and the other tangential to the buccal crown side respectively. We excluded third molars due to their low frequency and the occurrence of shape deviations. In addition, three standard dental indices were calculated [31]: 1) Crown module CM = (mesiodistal diameter + buccolingual diameter)/2; 2) Crown index CI = (100 x buccolingual diameter)/ mesiodistal diameter; and 3) Crown area CA = mesiodistal diameter x buccolingual diameter. The software MeshLab (v.2021.05) [29] was used to obtain dental measurements in the 3D models by means of the Measuring Tool. The dental measurements were obtained by G.Y in upper and lower teeth (I1, I2, C, P3, P4, M1 and M2, N = 28) on both sides, but only the right one was used in the statistical analyses. However, if a right tooth was missing the corresponding left antimere was used instead. This procedure helps to reduce statistical noise related to multicollinearity given the high antimere tooth size correlation [14]. The measurements of maxillary and mandibular teeth (N = 28) of 50 randomly selected 3D models were obtained twice to evaluate intraobserver consistency through intraclass correlation coefficients (ICC). In some cases, teeth show extensive damage due to pathologies (e.g., cavities), dental restorations, and heavy wear, which produces random missing data incompatible with multivariate statistical analysis. In the present study the average rate of missingness in the complete dataset was 0.038 and the missingness rate per variable ranges from 0.014 to 0.168. To solve this, we used the supervised machine learning algorithm “random forest” (N = 500 maximum number of iterations and 1000 trees) to impute missing data using the R package missForest v. 1.5 [32]. In S1 Fig a graph of the missing data is presented obtained through the R package naniar v.0.6.1 [33]. Raw dental measurements were size-adjusted and converted into shape variables by using the procedure recommended by Jungers et al. [34] which divides each measurement by the geometric mean (GM) for all the measurements for each individual. The GM was computed as the *n*th root of the product of the *n* variables [34]. Given that much of the odontometric differences between males and females are related to size rather than shape, this procedure reduces inter-individual dental size differences related to sexual dimorphism. In total, we obtained 98 dental variables in the Colombian sample investigated (28 raw measurements, 28 size-adjusted variables, and 42 indexes) which were included in all statistical analyses.

Regarding the comparative samples, we used the BL and MD diameters for all available teeth, excluding third molars. All measurements were recorded using a digital sliding caliper accurate to 0.01 mm. T.H. quantified his level of intra-observer error by separately re-measuring a Japanese sample, which was found to be negligible [12]. Because the differential preservation of the archaeological samples integrating the comparative sample investigated, some samples and variables presented relatively high rates of missing data. In such cases the imputation method mentioned above was used to generate complete datasets. Prior to imputation, individuals with more than 50% of missing data were removed from all subsequent analyses. Likewise, the same method outlined above was used to control for size reducing the overall effect of sexual dimorphism on tooth size, especially taking into account that the comparative dataset is mostly composed to males.

### Statistical analyses

All statistical analyses were performed in R version 4.1.0 [35]. Normal distribution of crown measurements was evaluated through a Shapiro-Wilk test. The pattern of intertrait correlation among the 28 measurements was evaluated through a cluster analysis and a correlation plot based on the Spearman correlation matrix using the package pheatmap v.1.012 [36]. To evaluate the effect of covariates -genomic ancestry, age, sex, and height- on dental measurements, we used partial correlation analysis using the package pysch v.2.2.9 [37]. All of these covariates are currently available in the population sample investigated. In addition, the percentage of sexual dimorphism was calculated as [(M—F)/F] x100 following Garn and colleagues [38], where M and F are the mean values for each of the 28 measurements of males and females, respectively. We also computed the percentage of sexual dimorphism in size-corrected variables and dental indices and in some cases the resulting differences will be negative when the females presented higher values than males. An independent Student *t*-test was used to evaluate the degree of sexual dimorphism using raw and size-corrected measurements as well as dental indices. The Bonferroni-adjusted *p* value threshold for significance was used *p*<0.0005 (0.05/100). To evaluate the phenotypic similarity/dissimilarity between Colombians, Mexicans and the continental populations two well-known multivariate exploratory techniques were applied, using the size-adjusted shape variables, including a principal component analysis (PCA) and a discriminant function analysis (DFA). PCA is a dimensionality-reduction technique that transforms a set of variables into smaller and uncorrelated ones retaining most of the information included in the dataset [39], while DFA is a statistical procedure that classifies unknown individuals and provides the probability of their classification into a certain group defined *a priori* [40]. For the PCA the variance-covariance matrix was used and for the DFA Mahalanobis *D*^*2*^ distances, K-fold cross-validated classification rates and the classification matrix were also obtained. A multidimensional scaling plot was computed from the *D*^*2*^ matrix in order to evaluate the biological affinities of the parental and Latin American samples investigated. Additionally, data points in the PCA and DFA analyses were tested to evaluate whether they contained any potential outliers. In each set of results, all the data points for one population group were taken, and their probability of being an outlier (i.e. outlierness) for that group was calculated using the LoOP method [41] implemented in the package DDoutlier [42]. Parameters k = 5 (number of nearest neighbours) and λ = 3 (a scaling factor) were taken following the authors’ recommendation. Points with a probability > 95% of being an outlier were excluded from the plots, and the 95% CIs were calculated after this removal. We also compute violin plots to describe the distribution of dental measurements using the using the package ggplot2 v3.4.1 [43].

### Estimation of continental ancestry

The individuals examined here have been previously genotyped on Illumina’s HumanOmniExpress or GSA chips [44–46]. After pruning for Linkage Disequilibrium, we retained 93,328 autosomal SNPs. Genotype data from the Colombian samples was merged with data for Africans and Europeans from 1000 Genomes [47] and data for selected Native American samples [48]. Individual admixture proportions were obtained using ADMIXTURE [49] using an unsupervised model.

## Results

### Biases control

The results of the dental wear analysis (S1 Table) showed that most of the sample exhibited low mean dental wear scores (range = 0.98 UP3–2.40 LM1) revealing its minimal impact on the odontometric analysis performed. The analysis of the virtual and physical dental casts (S2 Table) shows no systematic error, and the random error was below 1.5% with the exception of the mesiodistal diameter for the right lateral incisor (2.9%). These results confirm that there are not statistically significant differences between physical and virtual dental models. Likewise, the intraobserver error analysis (S3 Table) indicated high, positive and significant correlations between two measurement sessions, indicating observer consistency across the data collection. Of 28, only 4 measurements presented ICCs below 0.75 and the ICC mean was 0.84 ± 0.05. According to Koo and Li [50] scale, the overall reliability of the ICCs is good to excellent. Finally, the Shapiro-Wilk test (S4 Table) showed that the 28 dental measurements did not violate the assumption of normality and both the univariate and multivariate parametric analysis are not biased.

**Fig 1:**
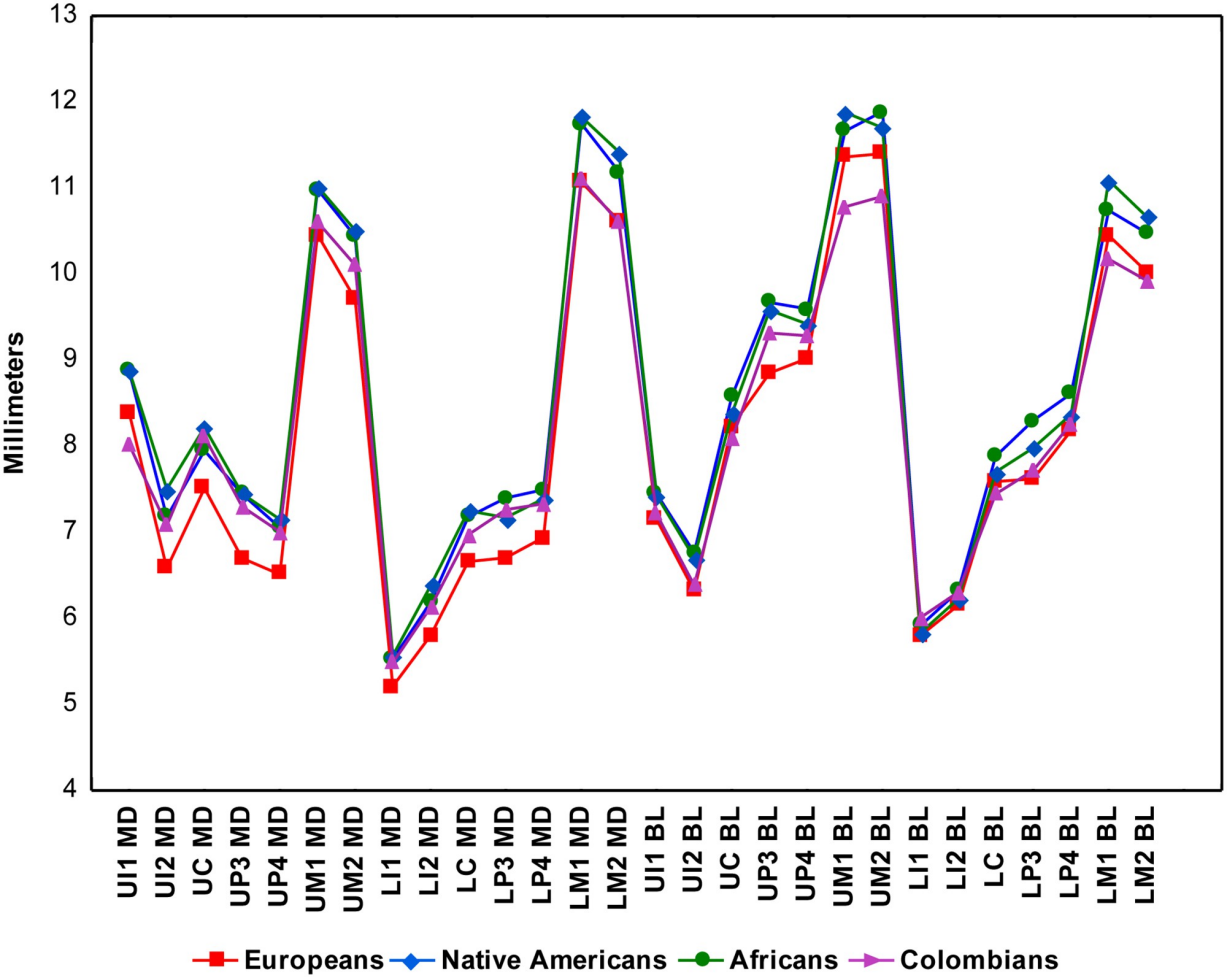
Line Plot of 28 MD and BL diameters (medians) in Colombians and three parental populations investigated.

**Fig 2:**
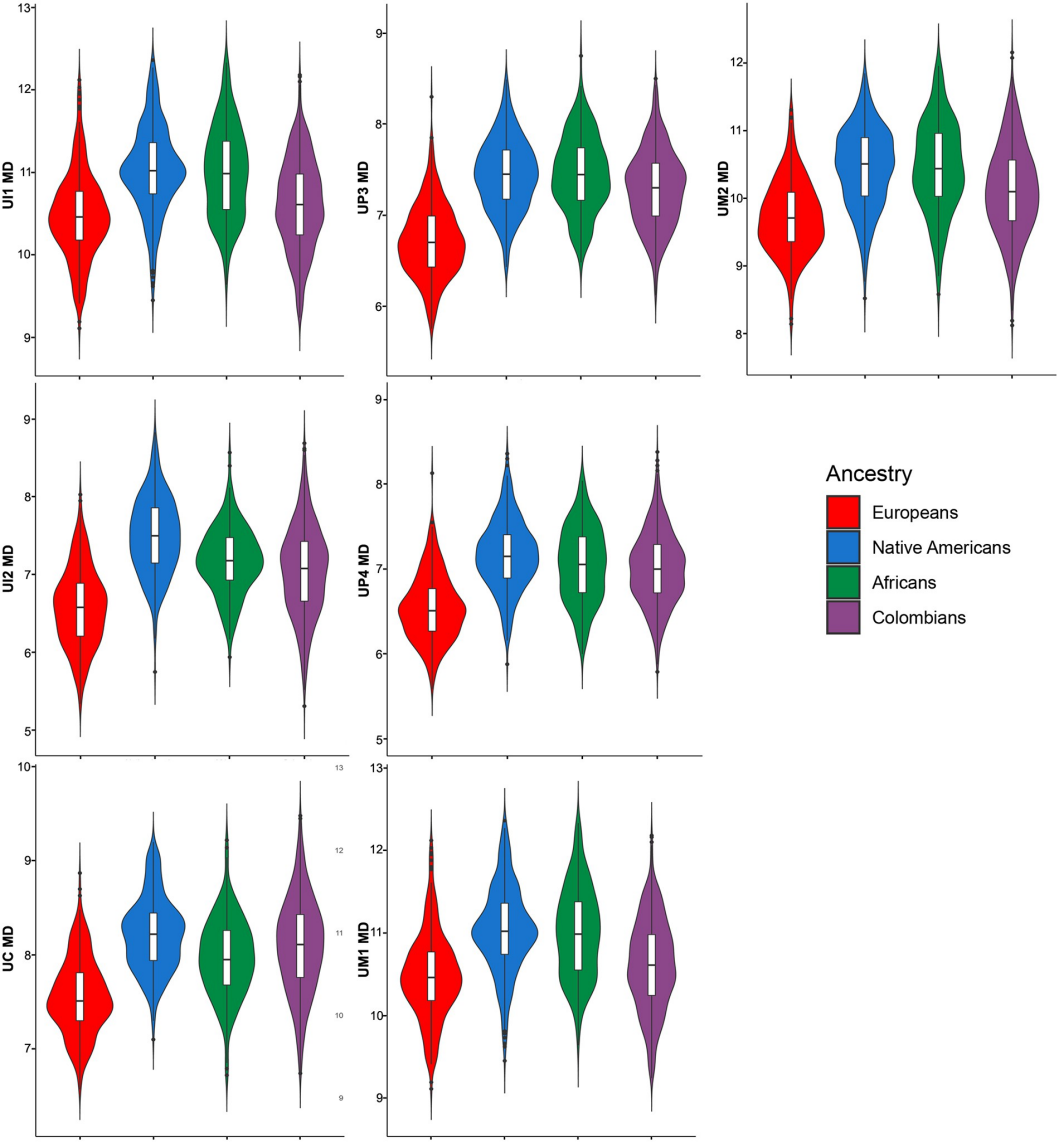
Violin plots of the MD diameters for the upper teeth in the Colombian population sample investigated.

**Fig 3:**
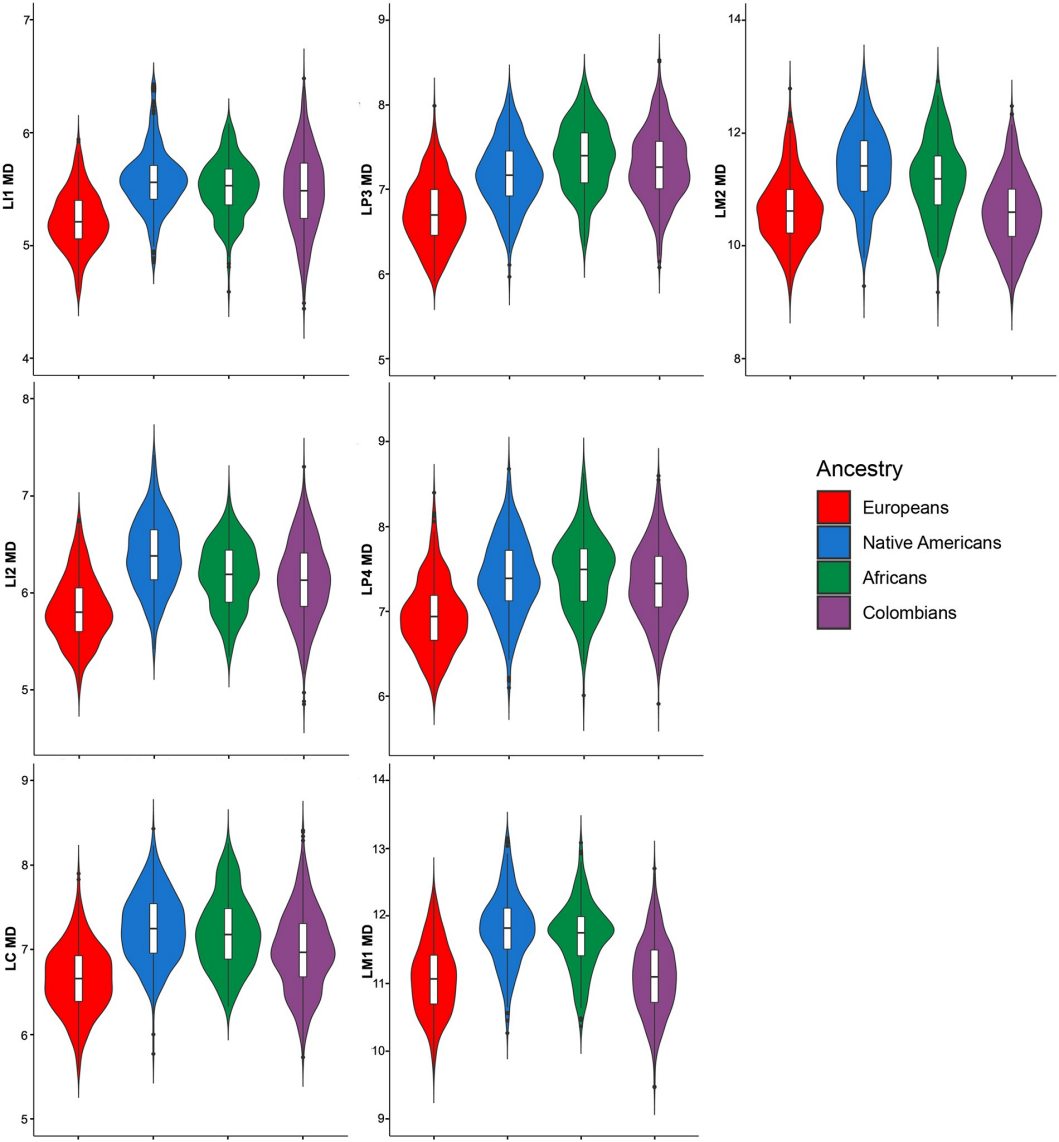
Violin plots of the MD diameters for the lower teeth in the Colombian population sample investigated.

**Fig 4:**
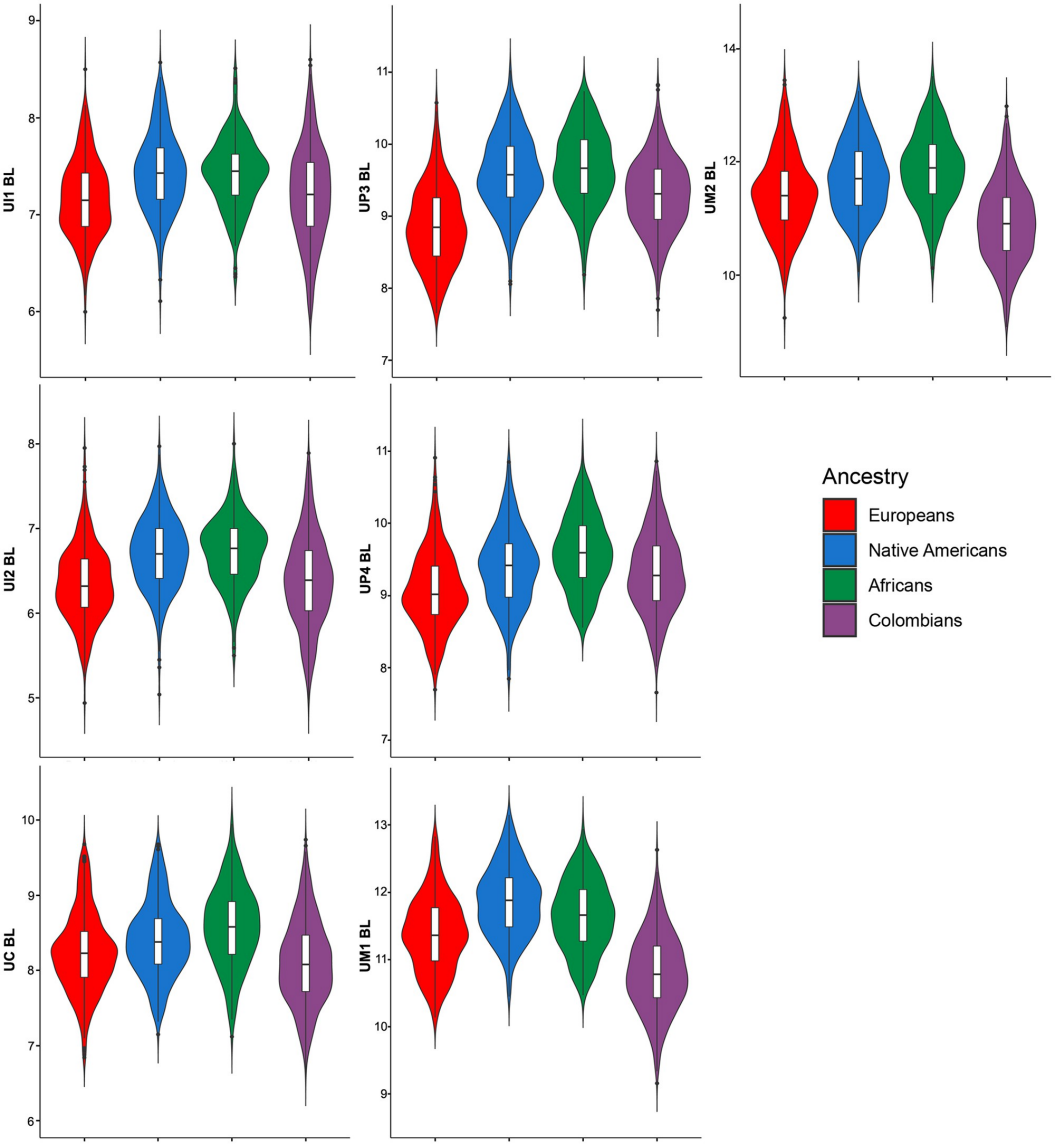
Violin plots of the BL diameters for the upper teeth in the Colombian population sample investigated.

**Fig 5:**
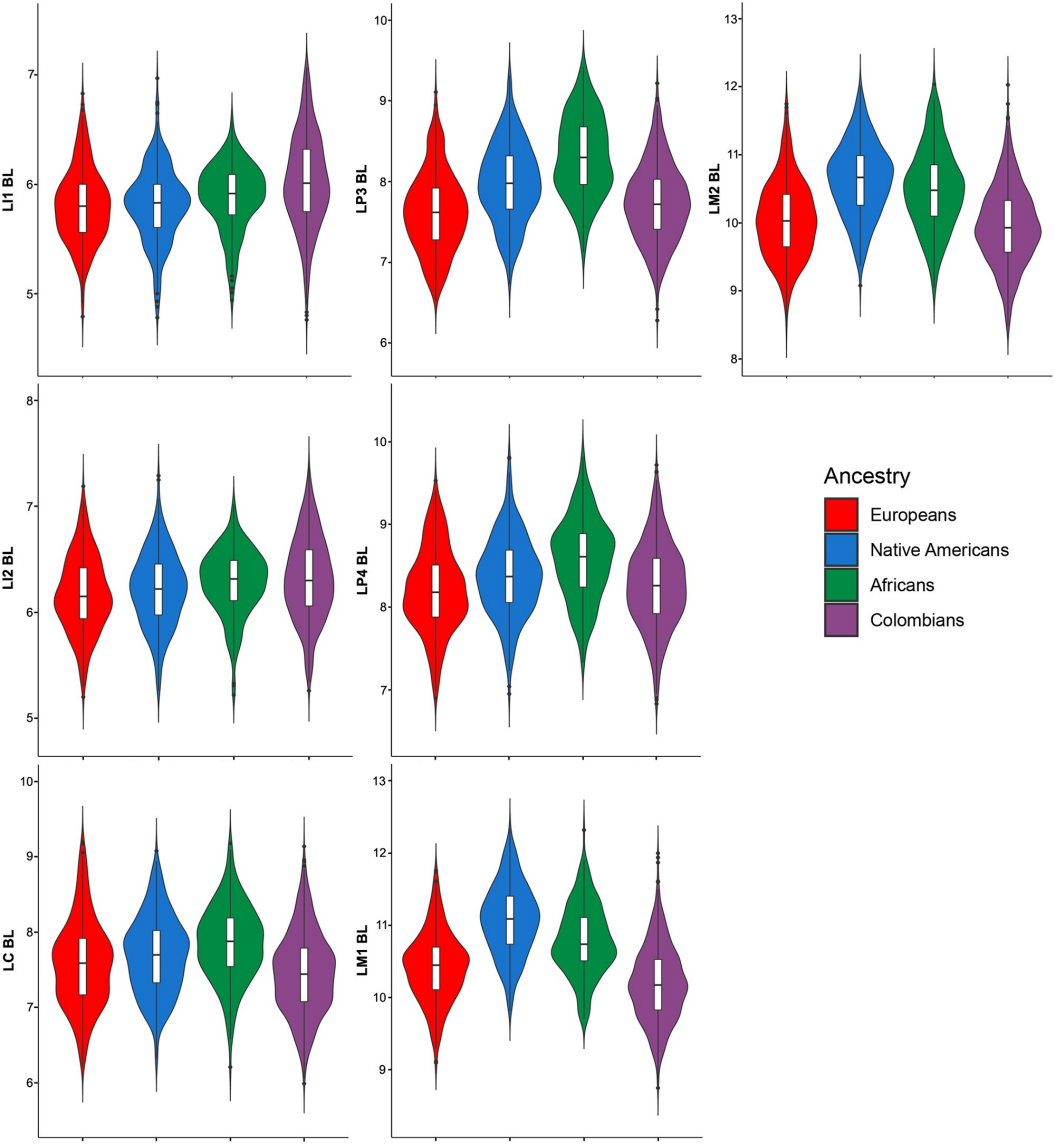
Violin plots of the BL diameters for the lower teeth in the Colombian population sample investigated.

The descriptive statistics for 28 dental measurements (raw) and three dental indices in Colombians (S5 and S6 Tables) and the parental populations (S7–S9 Tables) shows important differences. The medians for the 28 raw MD and BL diameters investigated show higher variation in Colombians than the parental populations according to measures of dispersion (standard deviations and coefficient of variation S5 and S6 Tables). Although the line plot of the medians (Fig 1) shows little differences between Colombians and parental populations, the violin plots for the MD (Figs 2 and 3) and BL diameters (Figs 4 and 5) show that Native Americans present the highest medians and interquartile ranges for most traits. Europeans usually have the lowest ones and exhibit more similarity to Colombians. Africans show intermediate values between Native Americans and Europeans and more differences with Colombians. The Colombian sample shares more similarities with Native Americans for the anterior/premolar measurements and with Europeans for molar measurements. This trend suggests a modular pattern of differentiation between Colombians and the parental populations involved in the admixture.

### Intertrait correlations

**Fig 6:**
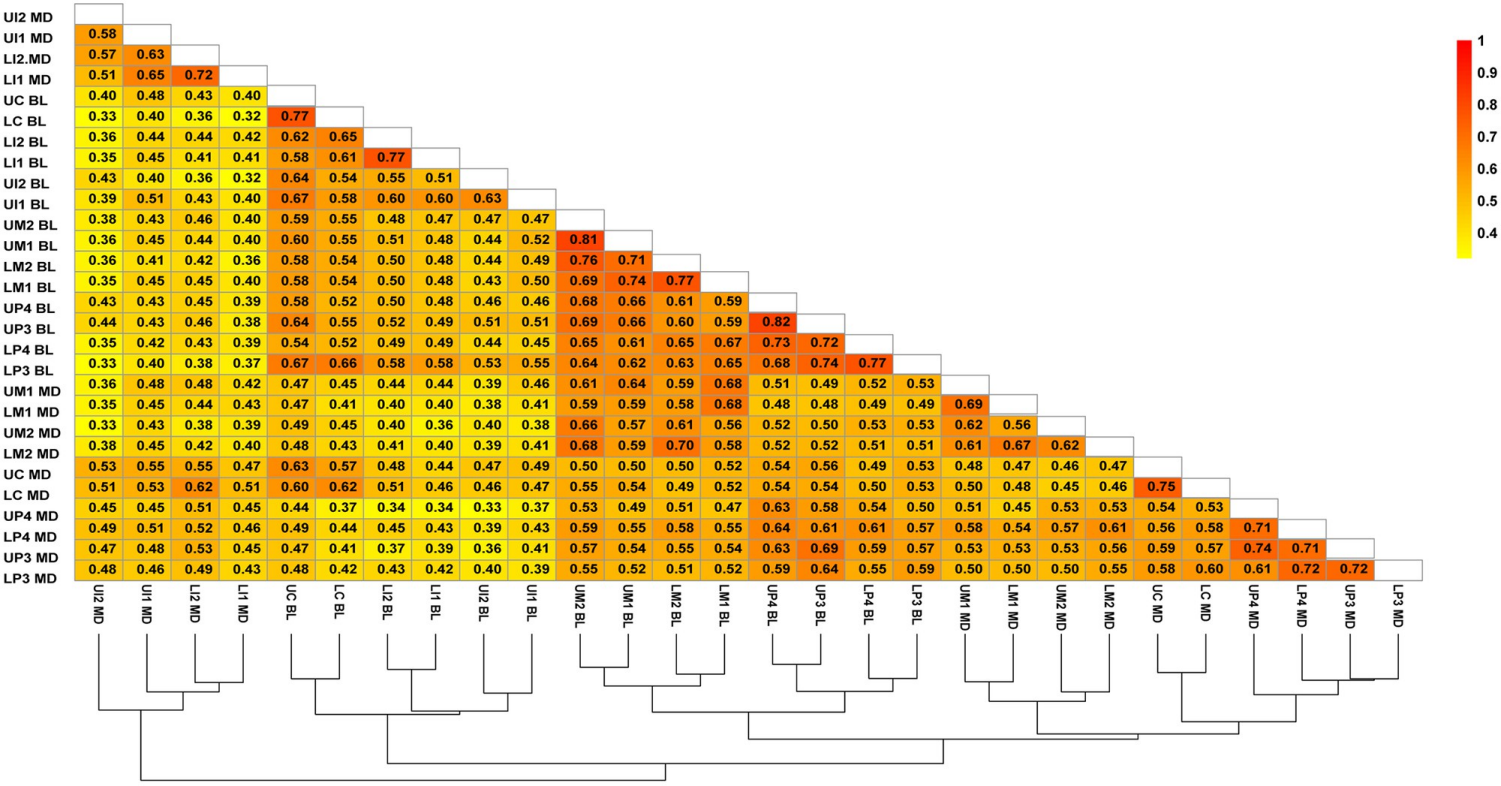
Correlation plot with hierarchical clustering showing Spearman correlations among the 28 MD and BL measurements used in the Colombian sample investigated.

The correlation matrix is represented as a correlation plot and a cluster analysis (Fig 6). The correlations among the 28 measurements are all positive, significant and higher than 0.32. The magnitude of the intertrait correlations is higher in the postcanine teeth (range = 0.8–0.4) than the anterior teeth (range = 0.7–0.3) and higher in the lower posterior teeth than in the upper postcanine dentition. A cluster analysis shows three main clusters, one of which is divided into three subclusters. The first cluster includes the MD diameter of the upper and lower incisors and is separate from the other two clusters. The second cluster includes the BL diameters of the upper and lower anterior dentition. The third cluster includes the BL and MD diameters of upper and lower molars, premolars and canines. This cluster includes three subclusters. The first subcluster includes the upper and lower canine and premolar MD diameters, although the canine measurements are separated from the premolar ones. The second subcluster includes the BL diameter of the upper and lower premolars and the third subcluster includes MD and BL diameters of the upper and lower molars. Within this subcluster the MD and BL diameters are well differented into separate subclusters. With the exception of the MD diameter of the upper and lower canines, overall this pattern of intertrait correlation suggests a modular structure of correlation where the anterior and postcanine dentitions tend to form distinct modules and there exists a clear difference between incisors, canines, premolars and molars.

### Sex-related variation

Significant differences were observed in dental size between males and females (Tables 1–6). The Student *t*-test results revealed that males exhibited larger tooth crown dimensions than females, although the degree of sexual dimorphism varied markedly and in some few cases females presented slightly larger tooth sizes than males (Tables 1–3). In addition, the variables investigated also exhibited distinct levels of sexual dimorphism. Males had significantly larger dimensions than those of females in 85.7% of the 28 raw measurements; in 28.5% of the 28 size-corrected measurements and in 78.5% of the 42 indices computed. Likewise, the percentage of sexual differences ranges between 1.3% and 6.9% for the raw measurements, between 0.0% and 3.6% for the size-corrected measurements and between 0.0% and 12% for the dental indices. Size-corrected variables and indices presented negative values indicating that females have higher values than males, that is, there is some shape and size variation between sexes. For raw measurements, BL diameters exhibited higher significant sexual differences than MD diameters, and the contrary pattern was observed for the size-corrected variables. Regarding dental indices, the CM and CA presented more significant values than CI. In all comparisons upper and lower canines exhibited the highest significance values and percentages of sexual dimorphism across all teeth. In addition, the mesiodistal diameter for the lower first molar and the buccolingual diameter for lower first premolar for the raw measurements presented the second highest and significant sexual differences. For the size-corrected variables, the mesiodistal diameter of the upper second premolar, upper lateral incisor and central and lateral lower incisors showed also important sexual differentiation. Finally, CM and CA for the upper and lower molars show a distinct difference between the sexes.

**Table 1:** Male and female differences in the Colombian sample investigated for 28 raw dental measurements (abbreviations as in the main text).

Tooth	Measure	Male mean (s.e)	Female mean (s.e)	Sex difference %	t-value	P-value
UI1	MD	8.9 ± 0.5	8.7 ± 0.5	2.2	4.24	**2.5E-5**
UI2	MD	7.1 ± 0.5	7.0 ± 0.6	1.4	0.86	0.389
UC	MD	8.3 ± 0.5	7.9 ± 0.4	5.0	9.11	**1.0E-6**
UP3	MD	7.4 ± 0.4	7.2 ± 0.4	2.7	5.11	**1.0E-6**
UP4	MD	7.0 ± 0.4	6.9 ± 0.4	1.4	2.90	0.039
UM1	MD	10.7 ± 0.5	10.4 ± 0.5	2.8	5.07	**1.0E-6**
UM2	MD	10.3 ± 0.6	9.9 ± 0.5	4.0	6.21	**1.0E-6**
LI1	MD	5.5 ± 0.3	5.4 ± 0.3	1.8	1.63	0.104
LI2	MD	6.2 ± 0.4	6.1 ± 0.3	1.6	1.39	0.165
LC	MD	7.2 ± 0.4	6.8 ± 0.4	5.8	9.51	**1.0E-6**
LP3	MD	7.3 ± 0.4	7.2 ± 0.4	1.3	4.14	**3.8E-5**
LP4	MD	7.4 ± 0.4	7.2 ± 0.4	2.7	4.19	**1.0E-6**
LM1	MD	11.3 ± 0.5	10.9 ± 0.5	3.6	7.70	**1.0E-6**
LM2	MD	10.9 ± 0.6	10.4 ± 0.6	4.8	7.85	**1.0E-6**
UI1	BL	7.3 ± 0.5	7.0 ± 0.5	4.2	6.38	**1.0E-6**
UI2	BL	6.5 ± 0.5	6.2 ± 0.5	4.8	6.65	**1.0E-6**
UC	BL	8.3 ± 0.5	7.9 ± 0.5	5.0	10.62	**1.0E-6**
UP3	BL	9.4 ± 0.5	9.1 ± 0.5	3.2	5.86	**1.0E-6**
UP4	BL	9.4 ± 0.6	9.1 ± 0.6	3.2	5.29	**1.0E-6**
UM1	BL	10.9 ± 0.5	10.6 ± 0.5	2.8	6.19	**1.0E-6**
UM2	BL	11.1 ± 0.7	10.7 ± 0.6	3.7	7.05	**1.0E-6**
LI1	BL	6.1 ± 0.4	5.9 ± 0.4	3.3	6.89	**1.0E-6**
LI2	BL	6.4 ± 0.4	6.2 ± 0.3	3.2	6.53	**1.0E-6**
LC	BL	7.7 ± 0.5	7.2 ± 0.4	6.9	13.21	**1.0E-6**
LP3	BL	7.8 ± 0.5	7.5 ± 0.4	4.0	7.98	**1.0E-6**
LP4	BL	8.3 ±0.5	8.1 ± 0.5	2.4	4.50	**1.0E-6**
LM1	BL	10.3 ± 0.5	10.0 ± 0.5	3.0	6.05	**1.0E-6**
LM2	BL	10.1 ± 0.5	9.8 ± 0.5	3.0	6.16	**1.0E-6**

Significant differences (p<0.0005) are in bold.

**Table 2:** Male and female differences in the Colombian sample investigated for 28 size-corrected dental measurements (abbreviations as in the main text).

Tooth	Measure	Male mean (s.e)	Female mean (s.e)	Sex difference %	t-value	P-value
UI1	MD	1.083 ± 0.05	1.089 ± 0.04	-0.5	2.30	0.021
UI2	MD	0.861 ± 0.05	0.876 ± 0.05	-1.7	4.35	**1.6E-5**
UC	MD	1.009 ± 0.04	0.997 ± 0.04	1.2	3.66	**2.7E-4**
UP3	MD	0.897 ± 0.03	0.903 ± 0.03	-0.6	1.78	0.075
UP4	MD	0.854 ± 0.04	0.867 ± 0.04	-1.5	3.92	**9.6E-5**
UM1	MD	1.302 ± 0.04	1.308 ± 0.05	-0.4	2.55	0.010
UM2	MD	1.246 ± 0.05	1.243 ± 0.05	0.2	0.58	0.563
LI1	MD	0.672 ± 0.03	0.685 ± 0.03	-1.9	4.70	**3.0E-6**
LI2	MD	0.752 ± 0.03	0.765 ± 0.03	-1.7	5.34	**1.0E-6**
LC	MD	0.875 ± 0.03	0.857 ± 0.03	2.1	4.88	**1.0E-6**
LP3	MD	0.896 ± 0.03	0.904 ± 0.03	-0.9	3.05	0.002
LP4	MD	0.906 ± 0.03	0.912 ± 0.03	-0.6	2.72	0.006
LM1	MD	1.370 ± 0.05	1.368 ± 0.06	0.1	0.08	0.937
LM2	MD	1.318 ± 0.05	1.309 ± 0.05	0.6	1.90	0.057
UI1	BL	0.891 ± 0.05	0.886 ± 0.04	0.5	1.15	0.248
UI2	BL	0.790 ± 0.03	0.782 ± 0.05	1.0	2.59	0.009
UC	BL	1.011 ± 0.04	0.990 ± 0.04	2.1	6.57	**1.0E-6**
UP3	BL	1.140 ± 0.04	1.143 ± 0.04	-0.2	1.59	0.113
UP4	BL	1.138 ± 0.05	1.140 ± 0.05	-0.1	1.49	0.136
UM1	BL	1.328 ± 0.04	1.333 ± 0.04	-0.3	1.05	0.296
UM2	BL	1.349 ± 0.05	1.339 ± 0.05	0.7	1.55	0.120
LI1	BL	0.747 ± 0.04	0.746 ± 0.03	0.1	1.94	0.052
LI2	BL	0.782 ± 0.03	0.782 ± 0.03	0.0	0.66	0.511
LC	BL	0.936 ± 0.04	0.903 ± 0.04	3.6	9.59	**1.0E-6**
LP3	BL	0.956 ± 0.04	0.950 ± 0.03	0.6	-2.65	0.008
LP4	BL	1.015 ± 0.04	1.023 ± 0.04	-0.7	2.04	0.041
LM1	BL	1.252 ± 0.04	1.259 ± 0.04	-0.5	1.78	0.075
LM2	BL	1.231 ± 0.04	1.233 ± 0.04	-0.1	0.45	0.652

Significant differences (p<0.0005) are in bold.

**Table 3:** Male and female differences in the Colombian sample investigated for 3 dental indexes (abbreviations as in the main text).

Tooth	Index	Male mean (s.e)	Female mean (s.e)	Sex difference %	t-value	P-value
UI1	CM	8.121 ± 0.02	7.916 ± 0.02	2.5	6.37	**1.0E-6**
UI2	CM	6.803 ± 0.02	6.652 ± 0.02	2.2	4.36	**1.4E-5**
UC	CM	8.317 ± 0.02	7.938 ± 0.02	4.7	11.67	**1.0E-6**
UP3	CM	8.404 ± 0.02	8.212 ± 0.02	2.3	6.11	**1.0E-6**
UP4	CM	8.254 ± 0.02	8.097 ± 0.02	1.9	4.76	**2.3E-6**
UM1	CM	10.844 ± 0.02	10.604 ± 0.02	2.2	6.52	**1.0E-6**
UM2	CM	10.705 ± 0.03	10.369 ± 0.02	3.2	7.70	**1.0E-6**
LI1	CM	5.830 ± 0.01	5.697 ± 0.01	2.3	5.49	**1.0E-6**
LI2	CM	6.290 ± 0.01	6.172 ± 0.01	1.9	4.90	**1.0E-6**
LC	CM	7.453 ± 0.02	7.043 ± 0.02	5.8	13.48	**1.0E-6**
LP3	CM	7.628 ± 0.02	7.420 ± 0.01	2.8	7.18	**1.0E-6**
LP4	CM	7.891 ± 0.02	7.733 ± 0.02	2.0	4.99	**1.0E-6**
LM1	CM	10.805 ± 0.02	10.529 ± 0.02	2.6	7.85	**1.0E-6**
LM2	CM	10.459 ± 0.03	10.140 ± 0.02	3.1	7.95	**1.0E-6**
UI1	CI	0.826 ± 0.00	0.815 ± 0.00	1.3	2.54	0.011
UI2	CI	0.928 ± 0.00	0.895 ± 0.00	4.8	5.14	**1.0E-6**
UC	CI	1.009 ± 0.00	0.998 ± 0.00	1.1	2.17	0.030
UP3	CI	1.277 ± 0.00	1.276 ± 0.00	0.0	0.35	0.727
UP4	CI	1.339 ± 0.00	1.326 ± 0.00	0.9	2.42	0.015
UM1	CI	1.021 ± 0.00	1.017 ± 0.00	0.3	1.19	0.234
UM2	CI	1.081± 0.00	1.078 ± 0.00	0.2	0.48	0.631
LI1	CI	1.120 ± 0.00	1.088 ± 0.00	2.9	4.75	**2.5E-6**
LI2	CI	1.047 ± 0.00	1.023 ± 0.00	2.3	4.03	**6.2E-5**
LC	CI	1.081 ± 0.00	1.060 ± 0.00	1.9	3.72	**2.1E-4**
LP3	CI	1.074 ± 0.00	1.054 ± 0.00	1.8	4.02	**6.3E-5**
LP4	CI	1.128 ± 0.00	1.125 ± 0.00	0.2	0.44	0.661
LM1	CI	0.915 ± 0.00	0.920 ± 0.00	-0.5	1.49	0.135
LM2	CI	0.936 ± 0.00	0.943 ± 0.00	-0.7	2.01	0.045
UI1	CA	65.43 ± 0.38	62.11 ± 0.33	5.3	6.50	**1.0E-6**
UI2	CA	46.33 ± 0.34	44.20 ± 0.30	4.8	4.59	**5.3E-6**
UC	CA	69.30 ± 0.39	63.11 ± 0.34	9.8	11.73	**1.0E-6**
UP3	CA	69.73 ± 0.38	66.57 ± 0.34	4.7	6.12	**1.0E-6**
UP4	CA	66.85 ± 0.39	64.41 ± 0.35	3.7	4.58	**5.4E-6**
UM1	CA	117.7 ± 0.59	112.6 ± 0.52	4.5	6.48	**1.0E-6**
UM2	CA	114.6 ± 0.68	107.5 ± 0.60	6.6	7.73	**1.0E-6**
LI1	CA	33.93 ± 0.20	32.45 ± 0.18	4.5	5.31	**1.0E-6**
LI2	CA	39.58 ± 0.22	38.13 ± 0.19	3.8	4.83	**1.6E-6**
LC	CA	55.58 ± 0.33	49.65 ± 0.29	12.0	13.43	**1.0E-6**
LP3	CA	58.21 ± 0.32	55.11 ± 0.28	5.6	7.14	**1.0E-6**
LP4	CA	62.16 ± 0.36	59.71 ± 0.32	4.1	4.97	**1.0E-6**
LM1	CA	116.6 ± 0.56	110.8 ± 0.49	5.2	7.80	**1.0E-6**
LM2	CA	109.4 ± 0.61	102.9 ± 0.54	6.3	7.94	**1.0E-6**

Significant differences (p<0.0005) are in bold.

**Table 4:** Partial correlations between raw dental measurements, age, sex, height and genomic ancestry in the Colombian sample investigated (abbreviations as in the main text).

Tooth	Measure	Age	Sex	Height	African	European	Native American
UI1	MD	0.003	**0.225**	0.023	0.138	**0.235**	0.134
UI2	MD	0.038	0.130	0.065	0.149	**0.236**	**0.220**
UC	MD	0.007	**0.357**	0.078	0.149	**0.287**	**0.214**
UP3	MD	0.016	**0.230**	0.018	**0.238**	**0.329**	0.136
UP4	MD	0.040	0.123	0.123	0.042	**0.250**	0.160
UM1	MD	0.020	**0.216**	0.173	0.091	0.081	0.022
UM2	MD	0.025	**0.308**	-0.015	0.104	0.139	0.021
LI1	MD	0.002	0.176	0.119	0.157	0.142	0.099
LI2	MD	0.054	0.134	0.187	0.157	**0.291**	**0.218**
LC	MD	0.005	**0.433**	0.069	**0.238**	**0.323**	0.146
LP3	MD	0.023	**0.217**	0.049	**0.235**	**0.221**	0.031
LP4	MD	0.035	**0.216**	0.086	0.126	**0.212**	0.072
LM1	MD	0.008	**0.327**	0.038	0.099	**0.215**	0.082
LM2	MD	0.100	**0.299**	0.026	0.107	**0.214**	0.048
UI1	BL	0.045	**0.243**	0.158	0.072	0.108	0.058
UI2	BL	0.004	**0.231**	0.026	0.080	0.115	0.015
UC	BL	0.101	**0.397**	0.074	0.159	0.148	0.051
UP3	BL	0.002	**0.291**	0.066	0.110	**0.234**	0.076
UP4	BL	0.006	0.198	0.201	0.026	**0.220**	0.086
UM1	BL	0.012	**0.269**	0.096	0.058	0.054	0.020
UM2	BL	0.035	**0.299**	**0.291**	0.053	0.112	0.044
LI1	BL	0.025	**0.280**	0.122	0.094	0.060	0.027
LI2	BL	0.048	**0.257**	0.135	0.077	0.134	0.042
LC	BL	0.168	**0.504**	**0.238**	0.081	0.123	0.028
LP3	BL	0.005	**0.286**	0.169	0.115	**0.218**	0.055
LP4	BL	0.057	**0.232**	0.128	0.051	0.207	0.073
LM1	BL	0.005	**0.236**	0.191	0.043	0.127	0.087
LM2	BL	0.067	**0.256**	0.211	0.048	0.138	0.062

Significant correlations (p<0.0005) are in bold.

**Table 5:** Partial correlations between size-corrected dental measurements, age, sex, height and genomic ancestry in the Colombian sample investigated (abbreviations as in the main text).

Teeth	Measure	Age	Sex	Height	African	European	Native American
UI1	MD	0.035	0.026	-0.142	0.103	0.117	0.092
UI2	MD	0.060	0.092	-0.037	0.102	0.148	0.139
UC	MD	0.048	0.105	-0.039	0.042	0.136	**0.219**
UP3	MD	0.040	0.084	-0.178	**0.215**	**0.231**	0.110
UP4	MD	0.028	**0.224**	-0.001	0.027	0.099	0.141
UM1	MD	0.043	0.147	-0.021	0.114	0.185	0.100
UM2	MD	0.068	0.016	-0.187	0.010	0.059	0.091
LI1	MD	0.054	0.108	-0.015	0.047	0.010	0.043
LI2	MD	0.121	0.180	0.137	0.092	**0.224**	0.176
LC	MD	0.025	**0.266**	-0.099	0.182	**0.213**	0.077
LP3	MD	0.042	0.065	-0.176	0.142	0.067	0.072
LP4	MD	0.066	0.099	-0.022	0.089	0.083	0.007
LM1	MD	0.023	0.025	-0.067	0.014	0.077	0.034
LM2	MD	0.170	0.021	-0.088	0.033	0.039	0.002
UI1	BL	0.039	0.039	0.070	0.063	0.071	0.022
UI2	BL	0.014	0.066	-0.032	0.025	0.018	0.040
UC	BL	0.169	**0.227**	-0.088	0.055	0.075	0.080
UP3	BL	0.018	0.028	-0.140	0.105	0.107	0.034
UP4	BL	0.003	0.063	0.097	0.015	0.055	0.002
UM1	BL	0.001	0.070	-0.120	0.169	**0.262**	0.108
UM2	BL	0.035	0.063	0.201	0.065	0.082	0.031
LI1	BL	0.016	0.053	0.063	0.017	0.126	0.107
LI2	BL	0.066	0.005	0.037	0.008	0.033	0.029
LC	BL	0.172	**0.374**	0.195	0.057	0.111	0.113
LP3	BL	0.032	0.048	0.093	0.048	0.039	0.071
LP4	BL	0.116	0.112	0.056	0.039	0.014	0.001
LM1	BL	0.022	0.126	0.139	**0.218**	0.175	0.016
LM2	BL	0.062	0.042	0.127	0.120	0.091	0.055

Significant correlations (p<0.0005) are in bold.

**Table 6:** Partial correlations between dental indices, age, sex, height and genomic ancestry in the Colombian sample investigated (abbreviations as in the main text).

Tooth	Index	Age	Sex	Height	African	European	Native American
UI1	CM	0.045	**0.295**	0.130	0.126	**0.224**	0.116
UI2	CM	0.011	0.193	0.137	0.091	0.189	0.137
UC	CM	0.004	**0.340**	0.071	0.119	**0.227**	0.127
UP3	CM	0.072	**0.382**	0.077	0.182	**0.264**	0.099
UP4	CM	0.018	**0.235**	0.106	0.174	**0.267**	0.119
UM1	CM	0.025	**0.231**	**0.242**	0.070	0.149	0.061
UM2	CM	0.019	**0.315**	0.039	0.064	0.100	0.010
LI1	CM	0.007	**0.294**	0.157	0.110	0.207	0.103
LI2	CM	0.036	**0.253**	0.199	0.160	**0.217**	0.092
LC	CM	0.027	**0.411**	0.150	**0.217**	**0.266**	0.106
LP3	CM	0.072	**0.450**	0.164	0.180	**0.211**	0.037
LP4	CM	0.020	**0.285**	0.157	0.133	0.205	0.057
LM1	CM	0.039	**0.326**	0.102	0.143	**0.221**	0.082
LM2	CM	0.068	**0.307**	0.111	0.083	0.162	0.073
UI1	CI	0.058	0.001	**0.231**	0.142	0.151	0.115
UI2	CI	0.069	0.079	0.108	0.111	0.153	0.114
UC	CI	0.006	0.011	-0.016	0.039	0.095	0.153
UP3	CI	0.123	**0.222**	0.045	0.163	0.197	0.134
UP4	CI	0.038	**0.221**	-0.060	0.058	0.013	0.093
UM1	CI	0.030	0.057	0.048	0.070	0.159	0.072
UM2	CI	0.044	0.041	0.144	0.116	0.150	0.036
LI1	CI	0.054	0.109	0.016	0.049	0.010	0.047
LI2	CI	0.040	0.144	-0.090	0.069	**0.216**	0.179
LC	CI	0.072	0.187	0.073	0.144	0.174	0.070
LP3	CI	0.104	**0.312**	0.155	0.125	0.135	0.060
LP4	CI	0.032	0.104	0.054	0.084	0.073	0.045
LM1	CI	0.053	0.051	0.091	0.009	0.057	0.003
LM2	CI	0.167	0.085	0.142	0.150	0.133	0.016
UI1	CA	0.040	**0.292**	0.125	0.129	**0.224**	0.117
UI2	CA	0.010	**0.218**	0.132	0.092	0.188	0.134
UC	CA	0.001	**0.331**	0.077	0.113	**0.215**	0.114
UP3	CA	0.070	**0.378**	0.080	0.185	**0.271**	0.105
UP4	CA	0.020	**0.218**	0.113	0.177	**0.270**	0.124
UM1	CA	0.030	**0.227**	**0.250**	0.071	0.151	0.066
UM2	CA	0.020	**0.317**	0.041	0.065	0.103	0.012
LI1	CA	0.010	**0.272**	0.163	0.098	0.194	0.099
LI2	CA	0.030	**0.252**	0.199	0.161	**0.216**	0.091
LC	CA	0.030	**0.405**	0.160	**0.218**	**0.262**	0.104
LP3	CA	0.070	**0.447**	0.167	0.181	**0.211**	0.037
LP4	CA	0.020	**0.283**	0.165	0.136	0.208	0.059
LM1	CA	0.050	**0.314**	0.119	0.141	**0.219**	0.078
LM2	CA	0.070	**0.305**	0.111	0.082	0.161	0.074

Significant correlations (p<0.0005) are in bold.

Partial correlation analyses (Tables 4–6) corroborate the Student *t*-test results revealing that the 89.2% of the raw measurements (Table 4) presented positive and significant low/mean correlation coefficients (range = 0.216–0.504) with the exception of the MD diameter of UP4 and UI2 and LI2. Regarding the size-corrected variables (Table 5) a small percentage of crown dimensions (14.2%) presented positive and significant weak correlation coefficients (range = 0.224–0.374). Lastly, the indices CM (range = 0.193–0.450) and CA (range = 0.187–0.447) (Table 6) presented low/mean positive and significant correlation coefficients for all teeth, while the CI (range = 0.012–0.317) exhibited the 21.4% of the variables with low and positive significant correlations. Overall, the canines exhibited the highest positive correlation coefficients across all teeth with the exception of the CI for UC. Similarly, LC exhibited stronger correlations with sex than UC. The upper and lower molars (raw measurements) and UP4 (size-corrected measurements) are the second most dimorphic teeth. The crown indices displayed differences regarding the degree of sexual dimorphism, while the CA and the CM presented high positive and significant coefficients for all teeth with the exception of CM UI2, the CI exhibited mostly low positive and non-significant coefficients.

### Age-related variation

We observed little influence of age on crown dimensions in the Colombians examined (Tables 4–6). Despite most dental measurements presented very low and non-significant correlations with age at least five of them presented a bit high positive correlation coefficients including LC BL diameter for the raw measurements (r = 0.168), the UC and LC BL diameter (r = 0.169 and r = 0.172) and the LM2 MD diameter (r = 0.170) for the size-corrected variables and the LM2 crown index (r = 0.167).

### Tooth size and body size correlation

We found little evidence of a significant relationship between body size (height) and tooth size in the Colombian sample investigated (Tables 4–6). Five dimensions presented significant correlations including the BL diameters of the UM2 (r = 0.291; p<0.0001) and the LC (r = 0.238; p<0.0003) for the raw measurements, and the CM (r = 0.242; p<0.0002) and CA (r = 0.250; p<0.0001) for the UM1 as well as the CI for the UI1 (r = 0.231; p<0.0005). The remainder measurements, including the size-corrected ones (Table 5), presented low and nonsignificant correlations.

### Tooth size and genomic ancestry

**Fig 7:**
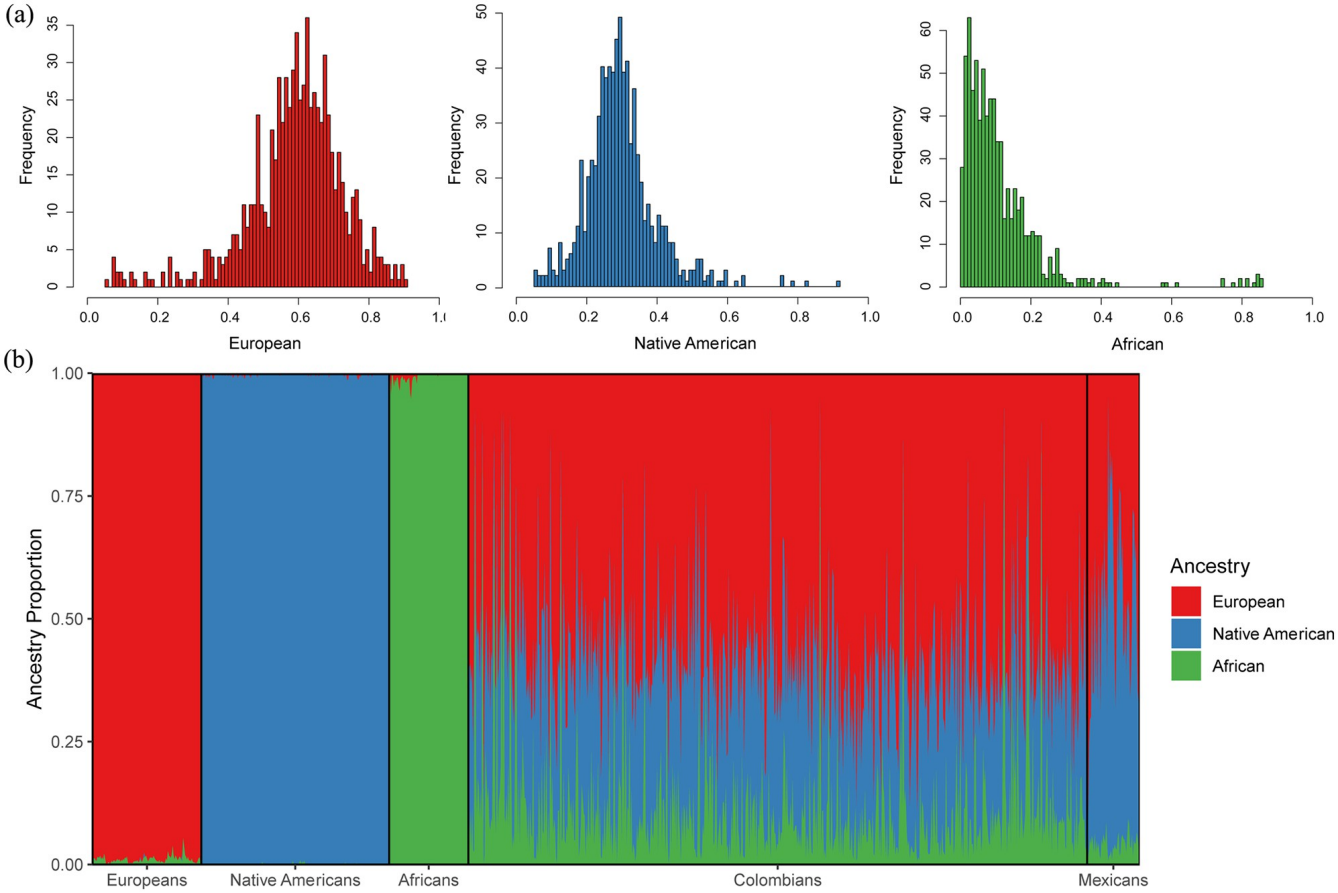
Distribution of individual European Native American and African ancestry using genome-wide SNP data in the Colombian sample (a). ADMIXTURE bar plot with the Native American, European and African admixture estimates for the Colombian and Mexican samples and three parental population samples (b).

Partial correlation analyses show a differential contribution of genomic ancestry to dental size variation in Colombians (Tables 4–6). Fig 7a shows the mean genomic ancestry proportions for the Colombian sample (0.59 European, 0.30 Native American, and 0.11 African) while Fig 7b shows the ancestry proportions in Colombians, Mexicans, and parental populations. For the raw dental measurements (Table 4), European ancestry presented the highest percentage of positive and significant correlations with dental size (50%; range = 0.212–0.329; mean = 0.261), followed by African ancestry (10.7%; range = 0.235–0.238; mean = 0.237) and Native American ancestry (10.7% range 0.214–220; mean = 0.217). MD and BL diameters of anterior teeth plus premolars presented the highest correlations with European ancestry. Only the MD diameter of the LM1 and LM2 exhibited significant correlations with European ancestry. African and Native American ancestries presented correlations with MD diameters of lower and upper incisors, canines and third premolars. The partial correlations between size-corrected measurements and genomic ancestry (Table 5) reveal a small number of positive and significant correlations with European (14.2%), African (7.1%) and Native American (3.5%) ancestries. With exception of MD diameters of lower canines and lateral incisors, European ancestry is correlated with BL and MD diameters of the posterior teeth. African ancestry is correlated with posterior teeth (UP3 MD and LM1 BL). Native American ancestry is only correlated with MD diameters of UC. Correlations between genomic ancestry and dental indices (Table 6) show that the European ancestry presented the highest correlations: 57.1% for CM (range = 0.211–0.267; mean = 0.237), CA (range = 0.211–0.271; mean = 0.236), and 7.1% for CI (LI2) in both anterior and posterior teeth. African ancestry showed significant correlations for CM and CA in lower canines, while the CI did not show correlation with African ancestry. Finally, no one index was correlated with Native American ancestry.

### Affinities between Colombians, Mexicans and parental populations based on dental size

The degree of dental similarity and diversity between Colombians, Mexicans and reference parental populations was explored through PCA and DFA using size-corrected variables. Furthermore, we compared the relatedness inferred from dental data to a PCA obtained from genome-wide SNP data.

### Principal component analysis

**Fig 8:**
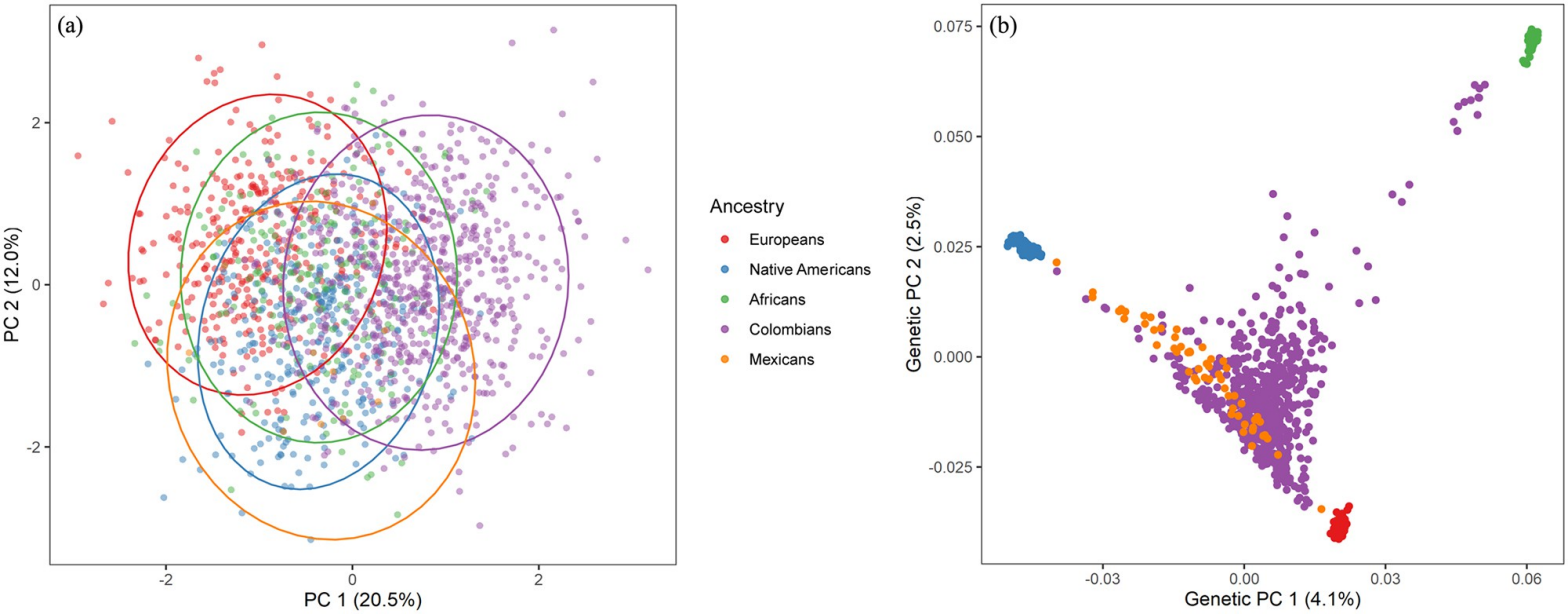
Scatterplot of the first two principal components (32.5% of the total variance) based on 28 MD and BL size-corrected dental measurements in two Latin American and three parental population samples. Ellipses of the 95% of confidence intervals are presented for the population samples investigated (a). Scatterplot of the first two principal components (6.6% of the total variance) based on genome-wide SNP data displaying the genetic relationships among two Latin American and three parental population samples (b).

The dental PCA (Fig 8a and Table 7) shows that along PC1 (20.5% of variance), most Colombians are characterized by positive scores and the parental populations as well as Mexicans are distributed along the left side of the plot with negative scores. These differences are not absolute as is shown by the 95% of confidence ellipses since some Africans, Europeans and Native Americans are also located on the right side of the plot and some Colombians occupy the left side. PC2 (12% of variance) suggests differences between Native Americans and Europeans where the former is mostly located at the bottom of the plot and the last mostly at the top. Africans have an unclear pattern and occupy a central position. Interestingly, Mexicans are closely related to Native Americans whereas Colombians have a more diffuse association with the parental populations, although they share more similarities with Europeans and Native Americans especially but also to Africans to a lesser extent. This pattern of dental-based population differentiation is reinforced by the 95% confidence ellipses. In S2 Fig the entire PCA data points including outliers are shown (S2A Fig), where outliers at two different probability thresholds, 95%, and 99%, are indicated. The genetic PCA (Fig 8b) 6.6% of the variance explained shows a pattern coincident with the dental PCA (Fig 8b), that is, Colombians share genetic similarities with Europeans while Mexicans are genetically related to Native Americans. Africans have a negligible impact in Mexican gene pool whereas some Colombians exhibited a substantial proportion of African ancestry. This pattern is not fixed since Colombians also share genetic similarities with Native Americans although Mexicans are distantly related to Europeans.

**Table 7:** Principal component analyses based on size-corrected measurements among three parental groups and two Latin American samples (abbreviations as in the main text).

Measure	PC1	PC2
UI1 MD	**0.420**	-0.156
UI2 MD	**0.446**	-0.247
UC MD	**0.488**	-0.045
UP3 MD	**0.503**	-0.335
UP4 MD	0.381	*-0*.*400*
UM1 MD	-0.251	*-0*.*424*
UM2 MD	-0.118	*-0*.*430*
LI1 MD	**0.408**	-0.207
LI2 MD	**0.400**	-0.248
LC MD	0.311	0.040
LP3 MD	**0.519**	-0.172
LP4 MD	0.342	-0.288
LM1 MD	-0.388	*-0*.*486*
LM2 MD	*-0*.*471*	*-0*.*501*
UI1 BL	-0.076	**0.415**
UI2 BL	-0.043	0.353
UC BL	-0.27	**0.608**
UP3 BL	0.174	0.056
UP4 BL	0.054	0.124
UM1 BL	*-0*.*810*	-0.039
UM2 BL	*-0*.*789*	0.045
LI1 BL	0.214	**0.487**
LI2 BL	0.112	**0.514**
LC BL	-0.271	**0.662**
LP3 BL	-0.182	0.322
LP4 BL	-0.148	0.252
LM1 BL	*-0*.*676*	-0.195
LM2 BL	*-0*.*641*	-0.153
Eigenvalue	0.0133	0.007
Total variance	20.552	11.989
Cumulative variance	20.552	32.542

High positive correlation coefficients (≥ 0.4) in bold; high negative correlation coefficients are in italics.

The dental-based affinities are associated with changes in several dental measurements (Table 7). PC1 exhibited high positive loadings (>0.4) for most upper and lower anterior teeth, including the first premolars for MD dimensions, whereas high negative loadings (>-0.4) were observed in the upper and lower first and second molars for MD and BL dimensions. Overall this pattern suggests that Colombians have a marked increase in the MD diameter of their anterior teeth and first premolars compared to their parental populations, mostly Europeans and Africans, which, in turn, have upper and lower first and second molars with increased MD and BL dimensions (Figs 2–5). Nevertheless, these differences are not absolute since Native Americans and Mexicans show a similar increase in their anterior tooth MD diameters. PC2 presented a similar trend regarding the anterior/post-canine dentition differentiation, that is, high negative loadings for the upper and lower first and second molars for MD dimensions and high positive loadings for the upper and lower anterior teeth for BL dimensions. This trend suggests that Native Americans have larger post-canine dentitions than Europeans and a relatively similar trend for anterior teeth (Figs 2–5). This pattern of morphological similarities/differences is not explained by sexual differences because much of the measures included do not exhibit significant sexual differences among the samples investigated for the size-corrected variables (Table 5).

### Discriminant function analysis

**Fig 9:**
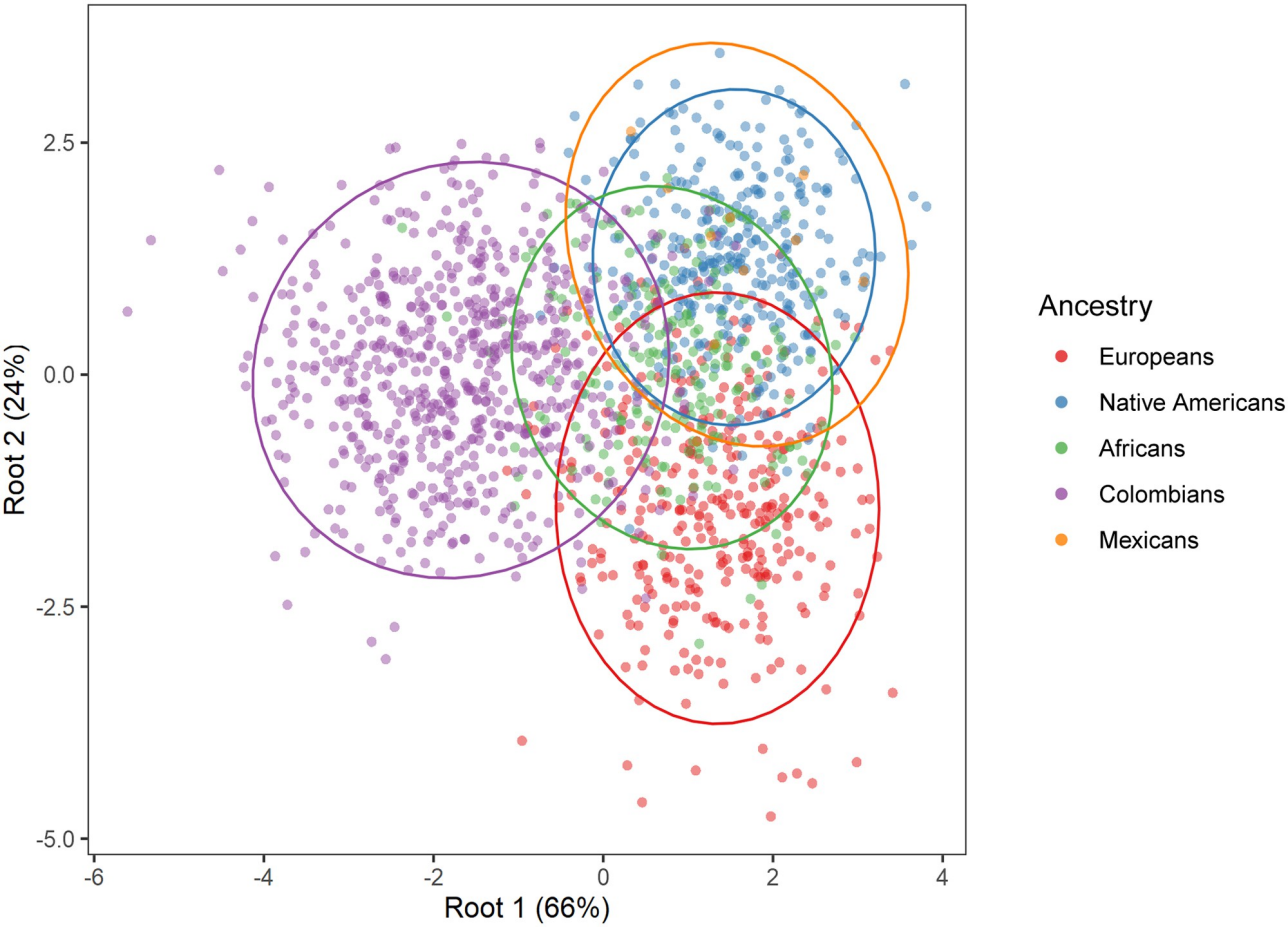
Scatterplot of the first two discriminant functions (roots) (84% of the total variance) based on 28 MD and BL size-corrected dental measurements in two Latin American and three parental population samples. Ellipses of the 95% of confidence intervals are presented for the population samples investigated.

**Fig 10:**
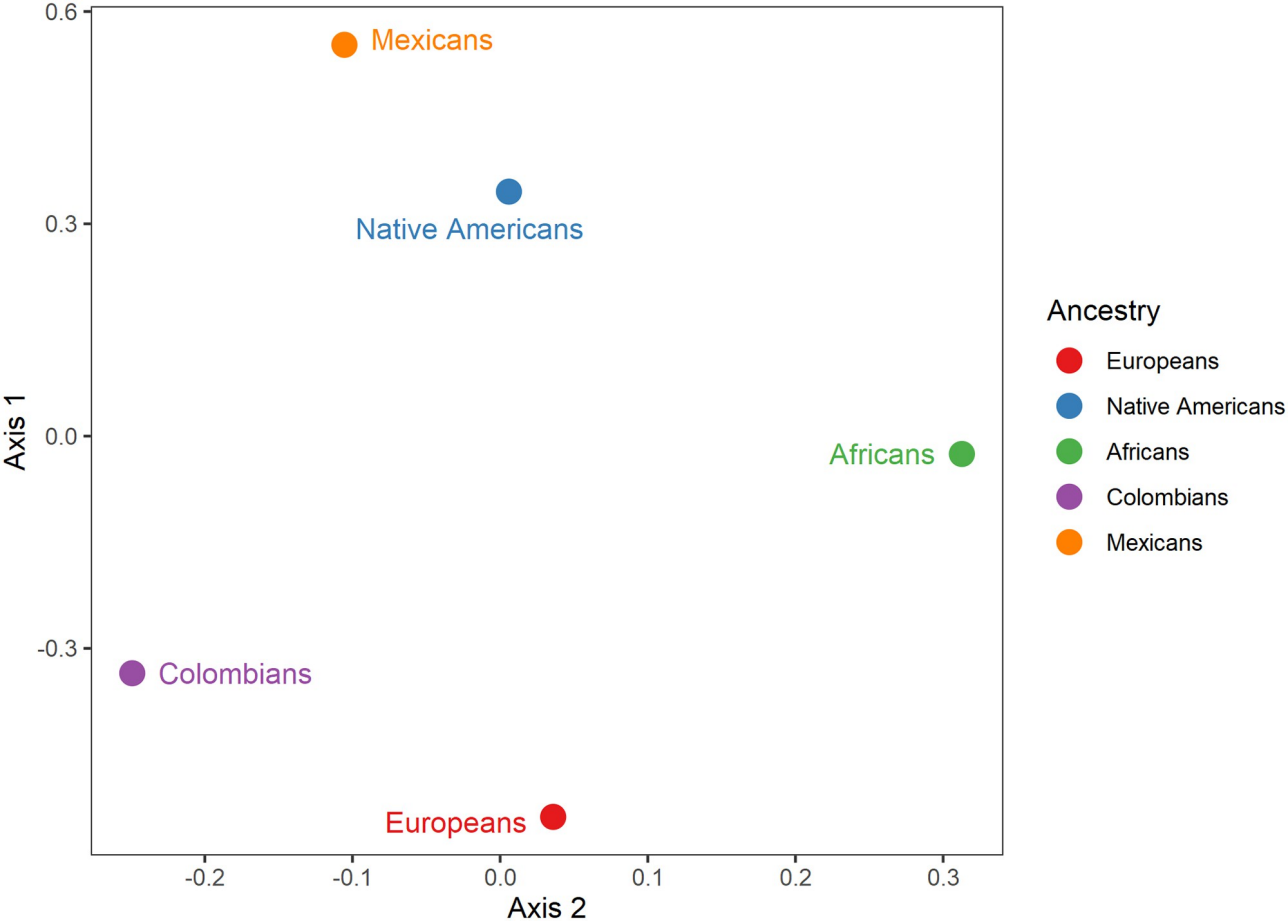
Multidimensional scaling plot based on Mahalanobis *D*^*2*^ distances exhibiting the phenotypic relationships among two Latin American and three parental population samples.

The results of the DFA show significant intergroup differences: Wilks’ Lambda: 0.13318 approx. *F* (112.6079) = 35.896 *p*<0.0001. The first two functions 90% of variance (Fig 9) show the degree of morphometric differentiation among Latin Americans and the reference parental populations. The first function (66% of variance) exhibits a distinction between Colombians and the reference parental populations including Mexicans; and the second function (24% of variance) shows a differentiation between Europeans and Native Americans. Africans present a central position and Mexicans are closer to Native Americans. In S2 Fig the entire DFA data points including outliers are shown (S2B Fig), where outliers at two different probability thresholds, 95%, and 99%, are indicated. Mahalanobis (*D*^*2*^) distances among the samples are statistically significant (Table 8), with the exception of the Native American- Mexicans pair. Importantly, Colombians and Europeans show a lower biological distance when Colombians are compared to Africans, Native Americans and Mexicans. A multidimensional scaling (MDS) plot based on the *D*^*2*^ matrix (Fig 10) shows a close similarity between Europeans and Colombians and Native Americans and Mexicans, while Africans remains distant from the other groups. Interestingly the MDS plot is very similar to the genetic PCA in both the inter-sample affinities and the positions of the groups. The classification matrix presents the observed and predicted cross-validated classifications for each individual into one of the four groups considered (Table 9). Since we used size-corrected variables that are minimally influenced by sexual dimorphism, we included both males and females in the analyses. The model correctly classified 83.6% of cross-validated cases. Africans presented the highest misclassifications (64.5%), whereas Europeans and Native Americas presented high correct classifications of 80.4% and 86.8%. Colombians presented the fewest misclassifications 6.9% (i.e., 93% of correct classifications). Remarkably, the observed and predicted classifications are similar indicating that the model is robust in terms of discrimination on the basis of dental size among the samples investigated. The classification results for Mexicans are not shown because 96% of the cases were incorrectly classified as Native American and two cases were classified as African (1.7%) and European (1.7%), respectively. This result is likely influenced by small sample size (N = 57) and/or the high proportion of Native American ancestry among Mexicans.

**Table 8:** Mahalanobis distances among three parental groups and two Latin American samples.

	Africans	Europeans	Native Americans	Mexicans	Colombians
**Africans**	0	5.6*	4.8*	6.8*	7.1*
**Europeans**	23.1	0	8.4*	11.1*	4.3*
**Native Americans**	21.6	43.6	0	2.6	6.7*
**Mexicans**	2.2	3.4	0.9	0	8.6*
**Colombians**	44.6	87.6	93.1	5.1	0

Above the diagonal *D*^*2*^ values, below of the diagonal F-values (28.15 df).

*Statistically significant at p<0.001

**Table 9:** Classification matrix derived from the DFA among four groups Africans, Europeans, Native Americans and Colombians (rows: Observed cross-validated classifications; columns: Predicted cross-validated classifications).

	% of correct classifications	Africans	Europeans	Native Americans	Colombians
**Africans**	64.5	133	29	23	27
**Europeans**	80.4	27	268	23	21
**Native Americans**	86.8	24	14	291	8
**Colombians**	93.1	20	20	23	748
**Total**	83.6	205	332	368	804

## Discussion

In the present study, we explored dental size variation in a large sample of living Latin Americans and assessed the influence of several factors, including age, sex, height (i.e., body size), and genomic ancestry. In addition, we explored the patterns of biological affinities between two admixed Latin American and three parental population samples. Our results show high dental size diversity among Colombians consistent with their mixed genomic ancestry, a differential contribution of the factors investigated to dental diversity and close biological relationships between Latin Americans and those parental populations that most contributed to their gene pool.

### Correlation patterns and dental size modularity

The pattern of intertrait correlations among Colombians is consistent with the modular variation observed among diverse primate taxa including fossil hominins [9, 51–53] and with the developmental modules predicted by the morphogenetic field and clone models [54–57]. Previous studies show that most hominins and other primates display anterior and postcanine modules, significant levels of correlation/covariation among the posterior dentition (premolars and molars), stronger integration in the mandibular dentition than in the maxillary dentition, and strongly integrated antagonist teeth (e.g., first molars) [52, 53, 58]. Likewise, recent analysis in modern humans reveal the important role of environmental factors (stress, diseases, etc) in the strength of morphological integration and high correlations between genetic and phenotypic covariance matrices [9, 52, 59].

Our results support these previous studies showing correlations at different levels, that is, between isomeres and tooth classes. Notably, in Colombians the phenotypic correlations among the postcanine dentition are higher than those observed for the anterior dentition indicating two modules that are relatively independent and internally integrated as well as stronger correlations for mandibular teeth [60]. The cluster of correlations suggests different morphological modules, with incisors and canines, relatively independent of premolars and molars and submodularity affecting premolars and molars. The pattern of correlations observed among admixed people: P3-P4, M1-M2 and P3-P4—M1-M2 coincides with those viewed in living and fossil hominins [52] and with the genetic/phenotypic correlations reported for recent humans, including African-Americans and European-Americans [9, 59, 60]. These findings also fully agree with the field and clone models of dental development of heterodont dentitions in mammals in general [54, 56] and modern humans in particular [55, 57]. The phenotypic integration detected in the present study can be explored from a genomic perspective in order to evaluate the level of genetic correlations (pleitropy) between distinct elements in the Latin American dentition.

### Sexual dimorphism in permanent dentition of Colombians

It is well documented that, on average males, have larger tooth crowns than females in contemporary humans, although the degree of sexual dimorphism varies within and between populations [11, 59, 61]. Accordingly, dental dimensions are widely used for sex estimation of human skeletal remains, an essential step in the reconstruction of the individual biological profile in bioarchaeological and forensic research [62, 63]. There are hundreds of studies addressing the degree and pattern of sexual dimorphism, from distinct dental elements and tissues in living and fossil hominins [11, 59, 61, 62, 64–66]. According to these and other studies, modern humans present moderate sexual dimorphism compared to other primates (e.g., great apes) and fossil hominins (e.g., australopiths) and the canine is the most sexually dimorphic tooth (i.e., 4–6%) depending of the group studied where the male mean canine dimension exceed those of females by 3 to 9% [11, 38, 67]. In addition, upper and lower first molar (deciduous and permanent) size usually displays significant levels of sexual dimorphism [11, 17, 63]. The degree of sexual dimorphism also differs among modern humans: Native Americans Ticuna from Colombia (range = 0.0% UM1–4.9% LC), Lengua from Paraguay (range = 0.7% UP4–7.5% UC), Pima from USA (range = 0.0%–6.3% LC) [11]; Africans Baka and Bantu-speakers from Central Africa (range 0.5% LP3–12.4% LC and 0.2% UP3–13.6% UC respectively) [7], African Americans USA (range = 0.0% LI1–8.5% LM1) and European Americans USA (range = 1.0% UI1–7.0% LC) [16].

Our results suggest that living Colombians exhibit moderate levels of sexual dimorphism in the permanent dentition. The percentage of sexual dimorphism (for raw measurements) in Colombians ranges from 1.3% LP3 to 6.9% LC, fairly similar to other less dimorphic populations such as Native Americans and European Americans [11, 16] and different from more dimorphic populations including Africans and Australian indigenous people [7, 68]. Size-corrected variables present lower percentages of sexual dimorphism (range = 0.1% LM2–3.6% LC), while dental indices presented higher differences (range = 0.0% UP3–12.0% LC). Interestingly, size-corrected variables and dental indices exhibited negative values which suggest that females presented higher values than males for some traits, although the differences are minor. Similar to other human populations and fossil hominins lower canines are the most dimorphic teeth across the arcade in Latin Americans. The moderate sexual dimorphism observed among admixed people can be explained by the nature of the ongoing dental evolution occurring in contemporary humans under a soft diet scenario (i.e., high consumption of ultra-processed foods) and the general absence of strong selective forces acting on tooth or body size. The little association between dental dimensions and body size in the study sample (Tables 4–6) suggest stability in the development of highly dimorphic traits among Latin Americans, especially when the male—female differences are standarized.

According to recent studies, dental tissues (enamel, dentine and pulp) contribute to the sexual difference we see in modern human teeth [16, 62]. While previous studies have suggested males have greater amounts of enamel that accounts for the differences between males and females [69], Schwartz and Dean [62] demonstrated that males have more dentine than females. In the Colombian sample, the male-female differences can be explained by multiple factors including the action of X-linked genes as previous studies have proposed based on individuals with chromosomal aneuploidy and with distinct mechanisms of morphogenesis [62, 70, 71]. Furthermore, authors have suggested that in modern humans the size differences among sexes are caused by the up-regulation of mitotic rates in response to elevated androgen levels in males, notably testosterone [72, 73]. Given the differences between Colombians and other recent human populations regarding the degree of sexual dimorphism, especially compared to Africans and European Americans, it is possible that development and mineralization times also influence the odontometric differentiation between Latin American males and females [16, 70]. In general, these results confirm previous investigations regarding the existence of moderate sexual dimorphism in the dentition of living humans where the canines (the lower ones especially) are the most dimorphic tooth. These observations are relevant for future forensic research.

### Age influence on tooth size among Colombians

Age estimation is an important step in personal identification in forensic research. Distinct morphological indicators and skeletal elements are used currently to assign age to human skeletal remains [74]. Teeth are key to age estimation through mineralization and eruption stages, enamel and root translucency and dental wear [75]. However, comparatively few studies have explored the use of dental measurements in age assessments in permanent dentition [76, 77]. For instance, Paulino and colleagues [77] detected a significant reduction of mesiodistal and buccolingual diameters in middle adults, especially in females compared to adolescents and young adults in a Spanish population sample. In addition, Murray et al detected a series of asymmetric age-related changes in teeth, including an important reduction in crown and root size in a sample of contemporary humans whose ages ranged from 10 to 60 years [76]. These changes include the increase of dentinal thickness and the decrease of odontoblast, subodontoblast and pulp fibroblast density, which are explained by the function of teeth and a general age-related decreasing density of different dental tissues. Overall these studies suggest that the crown and root size and minor crown components change according to age. The present study detected some weak correlations between age and raw dental measurements (LM2, UC MD and LC BL) and indices (LM2 CI). Despite the lack of statistical significance impose by the restrictive *p*-value used (*p*<0.0005) these results deserve some mention. Since some of the mentioned traits were positively correlated to sex, especially for the canines, it is possible that sexual dimorphism is also affecting the degree of age changes in tooth size observed in the study sample. The main explanation for the likely relationship between age and lower molar traits is that with age the interproximal wear tends to cause a reduction of the mesiodistal diameter. This is supported by previous studies of Native Americans and prehistoric Australians, which presented important reductions in tooth size according to their age, attributed mainly to high/mean rates of interproximal wear [78, 79]. Since the individuals investigated present ages that range from18 to 40 years and low to moderate dental wear rates this is a likely explanation. Interestingly, in a previous study of the same population sample, we found significant associations between dental nonmetric traits and age indicating that this is a probable factor accounting for a small portion of the observed diversity [80]. These results promote further investigation of the dental size/shape–age association using more detailed analyses including distinct crown components through 3D geometric morphometric methods.

### Tooth size and body size relationship

Previous studies have investigated the relationship between dental size and body size in several living and fossil hominoid taxa [81–83]. Comparatively, the population level has been less addressed, although some living and recent human population samples have been characterized indicating a differential relationship between body size and dental size among modern humans [82, 84–86]. In the present study we found that out of 98 variables only 5 presented significant correlations with height (a proxy of body size). Given the large and balanced sample size here investigated (N = 804, women/men = 446/358) these results are not biased by sample size. Four of these variables (BL UM2, BL LC, CM UM1 and CA UM1) also presented significant differences between sexes revealing a possible role of sexual dimorphism. Both molars and canines are very dimorphic teeth among modern humans and although we controlled for sex during the correlation analyses, raw measurements and indexes appear to reflect more strongly sexual differences. This result is similar to previous findings in other human populations investigated where the strength of correlation between body size and tooth size is highly influenced by sexual dimorphism [86] indicating a likely role of growth hormones. This inference could be partially supported by a recent study which indicated that growth hormones secretion is associated with tooth eruption and maturation [87]. In addition, Hikita and colleagues [88] found variants of the growth hormone receptor gene associated with changes in root and tooth length in some anterior teeth. However, to date, there is no direct evidence about the likely influence of genetic variants on both somatic growth and dental dimensions. It is also interesting that size-corrected variables do not presented correlations with body size suggesting that the association between body size and tooth size observed is mainly influenced by size rather than shape. Similar to a recent study [86] the correlation between dental size and body size observed among Colombians suggests sexual differences rather than a possible relationship between somatic growth and tooth development. This fact precludes a forensic use of this relationship in the population sample investigated since it is not possible to use dental dimensions to predict body size reliably if sexual dimorphism is not accounted for [85].

Finally, it is worth noting that despite the significant correlations found, the body size-tooth size covariation among living Colombians is weak as indicated by the low quantity of significant correlations (5%) suggesting that overall changes in body size are not linearly related to variations in dental measurements similar to other human populations adapted to distinct environmental scenarios [86].

### Tooth size and genomic ancestry in Colombians

Biological ancestry is a highly debated topic in biological and forensic anthropology and efforts have been made to standardize its use in studies that involve human skeletal remains [89–91]. Overall, global patterns of dental metric diversity mimic those viewed from craniofacial morphology and molecular variants, which have been interpreted as reflecting mostly population history, rather than natural selection [10–13, 60]. Accordingly, because tooth measurements vary according to expected patterns of neutral genetic data [10, 12], they have been used in biological ancestry assessments [13, 16, 18, 73]. However, currently the study of biogeographical origins of contemporary humans using tooth size is almost exclusively focused on populations with single continental ancestries, such as Africans, Europeans and Asians or populations samples classified using discrete categories such as European-Americans and African-Americans [13, 16, 59, 73, 92]. To date, few attempts have explored the use of dental metric traits to infer biological ancestry in admixed Latin American people (but see [18] the research for non-standardized dental measurements), and to our knowledge, this is the first time that the effectiveness of tooth dimensions to infer biological ancestry is assessed using individual proportions of genomic ancestry. Our results confirm previous investigations which suggest that dental measurements discriminate among human populations broadly distributed in a geographical sense, hence justifying their use in ancestry assessments [12, 13, 73]. In the case of the Latin American sample investigated, the European genomic ancestry presented the highest and strongest correlations with dental size in agreement with their higher proportion of European ancestry (~60%) and pattern of admixture (Fig 7). Remarkably, the investigation of dental nonmetric traits in this same population sample show exactly the same pattern of association [80], that is, higher correlations with traits reflecting European ancestry and close biological relationships with recent and contemporary Europeans, indicating that distinct features of the Latin American dentition can be used to infer biological ancestry and, at least in the sample investigated, both kinds of traits yielding similar estimations. The partial correlations (raw measurements and dental indices Tables 4 and 6), and the medians (Figs 1–5) show that invariably the dental metric traits significantly correlated with European ancestry (mostly third premolars and anterior teeth) display similar medians between Europeans and Colombians and differences with Native Americans and Africans. Remarkably, the PCA (Table 7) show that several of these traits characterize both Colombians and Europeans across the multivariate space who, in turn, share an overall reduction in tooth size compared to Africans and Asian/Native Americans. This pattern has been observed previously using global datasets [12, 13, 60].

A few traits were significantly correlated with African and Native American ancestries, but with the exception of LM1 MD, all presented higher correlations with European ancestry indicating that such ancestry presents the closer phenotypic similarities with present-day Colombians. Some of such traits (UC MD, LM1 MD and LI2 MD) present important differences in the parental population indicating likely “key traits” discriminating continental populations such as Europeans, Africans and Asian/Asian-descendants. This inference is supported by the continental odontometric differentiation detected by Kieser [11] and importantly, by the PCA, derived by Hanihara and Ishida [12] for uncorrected measurements that reveals remarkable continental differences in those measurements among modern humans. The correlation between size-corrected variables (Table 5) and genomic ancestries continue showing a strong association with the European genomic ancestry, but with the exception of LC and LI2 MD, all suggests a trend that involves postcanine tooth measurements (UP3 MD and UM1 BL). These traits also presented high PC loadings in the Hanihara and Ishida PCA [12], revealing their discriminatory power among continental populations. Particularly, UP3 MD is important to differentiate Eurasians, while UM1 BL differentiates Native Americans. Composite measures like the indices used also revealed that European genomic ancestry was the main ancestral component among Colombians.

Modern human differentiation in tooth size reflects mostly genetic differences given that previous studies have demonstrated that additive genetic effects account for ~60–90% of the observed variation [9, 93]; however, some population differences could also be related to the action of distinct types of natural selection [12, 13]. Despite the main odontometric differentiation viewed among continental populations was likely driven by random factors (i.e., genetic drift), dental size diversification observed between Latin Americans and parental populations could have been promoted by nonrandom factors (i.e., natural selection, phenotypic plasticity, etc.). In fact, a study in present-day Latin American populations (including the Colombian sample investigated here) detected strong signals of selection in genes involved in energy metabolism, likely as a result of dietary adaptations [94]. This could have important implications for the dental diversity among Colombians. The results of our previous research, which involved the investigation of very large datasets of admixed people, revealed extensive geographic variation in genomic ancestry across Latin America and its impact in biological diversity [24]. The highly diverse admixed Latin American dental morphology, a product of genomic ancestry and distinct biological and cultural factors, is then suitable to evaluate the efficacy of morphological indicators used in the reconstruction of the biological profile of unknown persons with non-homogeneous genomic ancestry distributions.

Overall, the present study through the investigation of individual proportions of genomic ancestry in a large sample of living Colombians confirm previous results indicating that dental size is useful in biological ancestry assessments in individuals of admixed ancestry [12, 13, 18]. These results are of special relevance in forensic contexts where more tools to estimate biological ancestry confidently are necessary especially in contemporary populations with admixed ancestry [91]. Here we expand upon previous efforts generating improvements in the ancestry assessment of individuals with mixed continental ancestry using tooth dimensions through the investigation of large datasets and genome-wide based ancestries.

### Biological relationships between Latin Americans and parental populations based on tooth size

One of the main objectives of the present research was to determining if tooth size is correlated with genomic ancestry proportions in a Latin American sample. Results indicate that several dental metric traits presented positive and significant correlations with genomic ancestry. However, since most studies assessing biological ancestry using dental dimensions and other features of human dentition do not incorporate genome-wide data in their comparisons, another important aim was to determine if there is a strong correspondence between genetically estimated ancestry and phenotypically-based affinities in order to extrapolate the present results to other human populations.

The results of the multivariate exploratory analyses (PCA and DFA) show that Mexicans and Colombians present distinct patterns of biological affinities, mostly reflecting their proportions of genomic ancestry. In the case of Colombians, the PCA and DFA show that Europeans are the phenotypically closest group, a result coincident with both their higher proportion of European genomic ancestry (~60%) and the percentage of significant correlations between dental traits and such ancestry. This is reinforced by the MDS plot based on Mahalanobis *D*^*2*^ distances (Fig 10 and Table 8) which show that Colombians and Europeans share close affinities. Likewise, Mexicans shared most of the morphospace with Native Americans in both PCA and DFA analyses. The MDS plot reveals a strong similarity between these samples and its *D*^*2*^ distance is non-significant and the lower one across all comparisons. The Mexican–Native American relationship is in full agreement with the proportions of genomic ancestry estimated from a large sample of contemporary Mexicans (N = 1622) 56% Native American, 35% European and 5% African (CANDELA consortium [24]). This suggests that contemporary Latin Americans, in this case represented by Colombians and Mexicans, exhibit close affinities with those parental populations that contributed the most to their genetic makeup, Europeans and Native Americans, respectively. Remarkably, the MDS plot (Fig 10) is in agreement with the genetic PCA plot revealing that both tooth size and genome-wide SNP data display a similar pattern of biological affinities between descendant and parental populations.

This study confirms that the affinities between Latin Americans and their parental population mostly reflect their proportions of genomic ancestry, an observation consistent with their population and demographic histories. We also corroborate previous studies indicating the usefulness of use of tooth size in differentiating continental populations [12, 13]. These results also support previous statements about the use of metric and nonmetric features of human dentition as proxies of neutral genetic data [8–10, 80, 93, 95], and as predictors of genomic ancestry [6, 80, 96].

The patterning of biological affinities among Latin American and their putative parental populations is related to explicit changes in the dentition, that is, an increase in the mesiodistal diameter in the Colombian anterior teeth and third premolars (although Mexicans and Native Americans tend to share this trend) compared to the parental populations which, in turn, present upper and lower first and second molars with increased MD and BL diameters. A similar trend regarding the anterior/posterior odontometric differentiation in modern humans was detected previously when a worldwide dataset was investigated [12]. Colombians also follows this pattern of modular variation indicating that despite admixture their dentition continues retaining patterns of variation that characterize modern humans and extinct hominins [52]. The stability in the degree of integration and the retention of ancestral patterns of modular variation in the Latin American dentition suggests that few strong evolutionary changes occurred during their recent history, however, minor changes like those observed here in tooth size suggest more diverse and heterogeneous dynamics at the microevolutionary scale. Previous studies have suggested that natural selection played a major role in modern human dental evolution [97, 98], while recent research highlights a pattern of diversity similar to neutral genetic variants [10, 12]. The patterning of anterior teeth/first premolars with increased MD diameters and second premolars and molars with decreased MD and BL diameters observed in Colombians could be linked to dietary adaptations like those detected in the same population related to strong signals of selection in genes involved in energy metabolism [94]. Changes in dietary habits (e.g., high consumption of carbohydrates and highly processed foods, etc.) [99], or alternatively, constraints in developmental timing caused by unpaired changes between teeth and jaws [100] could be responsible for the morphological differentiation between anterior and posterior teeth in present-day Colombians. However, other explanations are possible. It is thought that *EDAR* is involved in the overgrowth of mesial and distal marginal ridges of incisors and canines (i.e., shoveling), then increasing the mesiodistal diameter [70, 101, 102]. Kimura and colleagues [70] found that *EDAR* is correlated to distinct metric and nonmetric traits in Asian populations, and interestingly *EDAR* was also correlated to PC1-3 (60.12% of total variance) which describe overall tooth size, the ratio of MD to BL diameters and anterior versus posterior tooth sizes. Likewise, Park et al [101] found a highly significant association between *EDAR* and crown size, especially mesiodistal diameters of anterior teeth in Korean and Japanese populations. These findings suggest that *EDAR* could be one of the genetic factors influencing a number of phenotypic changes in the human dentition which includes a remarkable differentiation between anterior and posterior tooth sizes in Asian and Native American populations and those populations with substantial contributions of Asian/Native American ancestry like most Latin American populations. The similar trend exhibited by Native Americans and Mexicans detected in the present study also agrees with the likely influence of *EDAR* in the pattern of anterior/posterior differentiation in Asian-derived populations. Accordingly, the pleiotropic action of *EDAR* could explain partially the increase of MD diameter in the anterior dentition, and concomitantly, the MD and BL reduction in upper and lower molars observed in living Colombians. Future studies will clarify the role of *EDAR* and other genetic variants in the dental differentiation observed among modern humans at intra and inter-population levels and more specifically the finding of *EDAR* in the Colombian sample investigated will give more support to the hypothesis here outlined.

Apart from a forensic perspective, the percentages of classification obtained here (Table 9) support the use of dental metrics in biological ancestry assessments in unknown persons. On the basis of dental metrics, the correct observed and predicted cross-validated classifications for European (80.4%), Native Americans (86.8%) and Colombians (93.1%) were higher than those derived from previous studies investigating continental populations [13]. Africans presented the lowest correct classifications (64.5%) which is likely related to sample size bias given that the number of Africans included in the present study is lower than Europeans, Native Americans and Colombians. Despite this, the total percentage of correct classifications for admixed and continental population is relatively high (~84%) indicating the effectiveness and potential use of dental metric traits in forensic contexts.

Our results are in line with recent landmark-books and papers in forensic and biological anthropology which explored the weakness and the strengths of biological affinities and biodistances in forensic and bioarchaeological contexts [6, 103, 104]. Unfortunately, contemporary Latin Americans were not included or were subsidiarily investigated in those studies indicating that there is a general lack of knowledge about the biological diversity and affinities of admixed people and their potential use in forensic and biohistorical reconstructions. This and another related study [80] strongly supports the use of features of human dentition in assessments of age, sexual dimorphism, and biological ancestry among living Latin Americans. In particular, the present results suggest that biological affinities based on morphological traits are useful to differentiate populations and individuals with admixed ancestries. This is highly relevant in Latin America where there is high genetic and phenotypic diversity, and a better morphological characterization is required to improve methods in forensic identification.

## Conclusions

Our odontometric analysis of a large Latin American sample from Colombia explored the role of distinct factors underlying dental diversity, including age, sex, body size and genomic ancestry. We also investigated the pattern of trait association and the biological affinities, including cross-validated classifications, with three parental populations and two samples from Colombia and Mexico. Results suggest intertrait correlations at different levels, that is, between isomeres and tooth classes mostly reflecting different morphological modules. Tooth size in living Latin Americans is correlated to age, sex and body size for distinct variables in different teeth being the canine the most dimorphic tooth.

Our results corroborate the utility of dental metric traits to distinguish modern human populations and to relate individuals to those populations. This research revealed that the estimation of biological ancestry on the basis of dental metric traits is possible in Latin Americans with diverse genetic ancestries. Despite the lack of a clear continental differentiation in tooth size among modern humans our results suggest that the dental affinities of contemporary admixed Latin Americans are consistent with their individual genetic ancestry. This implies that tooth size can be used to infer continental ancestry in populations exhibiting a heterogeneous distribution of genomic ancestry. However, biological ancestry assessment using dental metric traits is still obscured by the lack of a detailed knowledge of the genetic architecture underlying dental development (i.e., the number of loci and allele frequencies at these loci, additive/dominant/recessive genetic effects) and by the distribution of individual genomic ancestry in the study sample. This suggest that further studies investigating paired samples, that is, including both morphometric and genetic variables in continental and local populations as well as in admixed people, are necessary to outline the genetic basis of dental metrics and to reveal their informativeness in biological ancestry assessments.

Finally, given the marked genetic and morphological differences observed between Mexicans and Colombians, the terms Hispanic or Latino frequently used in forensic and anthropological studies to refer to populations from Central and South America and the Caribbean has no biological sense and are inadequate to describe the wide biological diversity observed in those regions. A critical assessment of the population terminology used in current forensic and anthropological research to refer to contemporary Latin American populations is beyond the scope of this work. However, such an assessment would be beneficial to the development of a more accurate terminology, especially in the light of recent discussions concerning the use of the term *biological ancestry* in anthropological and forensic sciences [90, 91, 105]. Importantly, research to move beyond these terms is currently moving forward.

## Supporting information

S1 Fig Summary of missing data present in the dental data set for the Colombian sample investigated. (TIF)

S2 Fig The entire PCA (a) and DFA (b) data points including outliers are shown, where outliers at two different probability thresholds, 95%, and 99%, are indicated. (TIF)

S1 Table Mean, minimum and maximum wear scores for the Colombian sample investigated (abbreviations as in the main text). (DOCX)

S2 Table Paired *t*-test results and error percentage validating scanner reproducibility (abbreviations as in the main text). (DOCX)

S3 Table Intraclass correlation coefficients for 28 measurements in the Colombian sample investigated (abbreviations as in the main text). Significant differences are in bold.
(DOCX)

S4 Table Results of the Shapiro-Wilk test evaluating the assumption of normality for 28 MD and BL diameters investigated (abbreviations as in the main text). (DOCX)

S5 Table Descriptive statistics of 28 tooth crown raw measurements for the Colombian sample investigated (abbreviations as in the main text). (DOCX)

S6 Table Descriptive statistics of 3 dental indexes for the Colombian sample investigated (abbreviations as in the main text). (DOCX)

S7 Table Descriptive statistics of 28 tooth crown measurements for the Native American sample investigated (abbreviations as in the main text). (DOCX)

S8 Table Descriptive statistics of 28 tooth crown measurements for the European sample investigated (abbreviations as in the main text). (DOCX)

S9 Table Descriptive statistics of 28 tooth crown measurements for the African sample investigated (abbreviations as in the main text). (DOCX)
