# Supplementary figures and images for: Dental size variation in admixed Latin Americans: Effects of age, sex and genomic ancestry
Source: PLoS One. 2023 May 4;18(5):e0285264. doi: 10.1371/journal.pone.0285264 (PMC10159210; doi:10.1371/journal.pone.0285264)

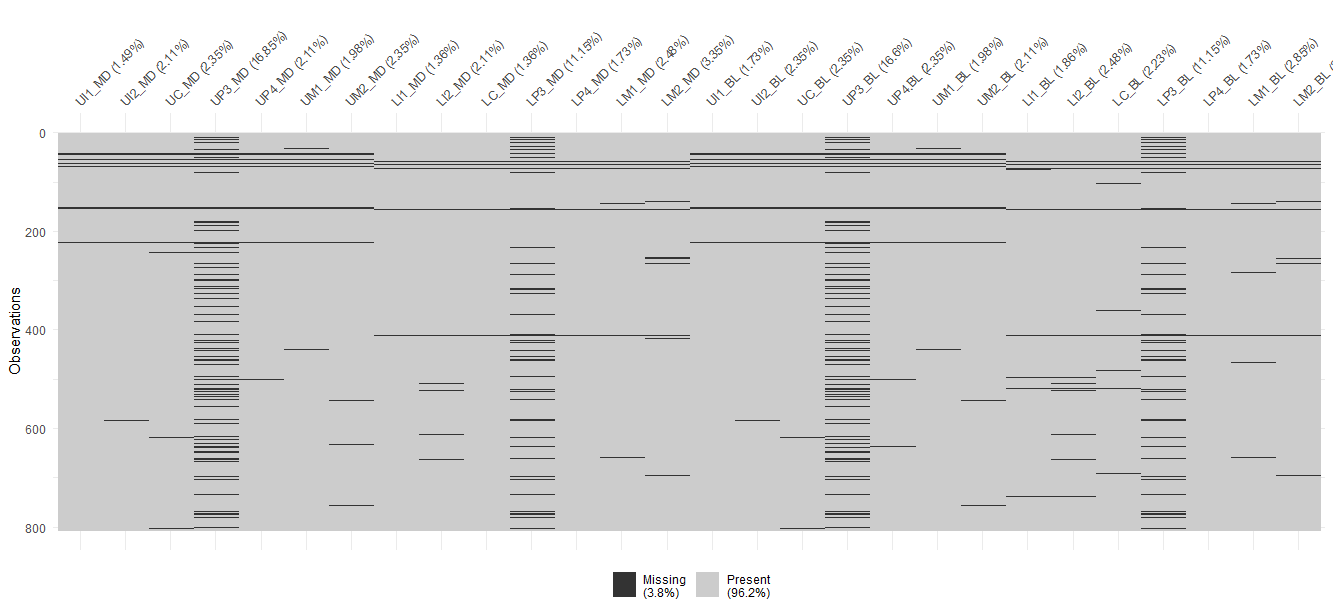

Supplement: S1 Fig — (TIF) [file pone.0285264.s001.tif]

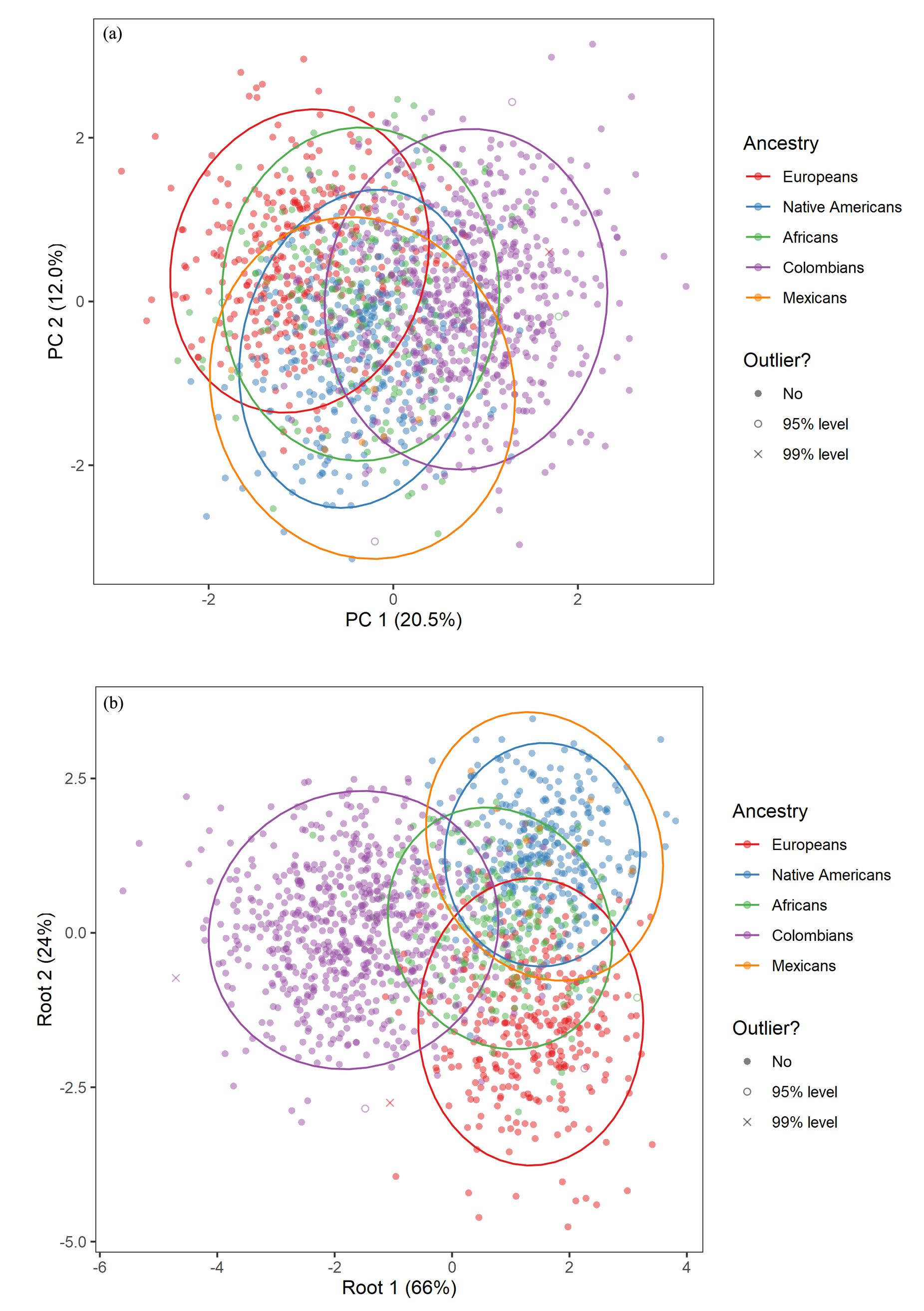

Supplement: S2 Fig — (TIF) [file pone.0285264.s002.tif]
