# Supplementary material for: Dental size variation in admixed Latin Americans: Effects of age, sex and genomic ancestry
Source: PLoS One. 2023 May 4;18(5):e0285264. doi: 10.1371/journal.pone.0285264 (PMC10159210; doi:10.1371/journal.pone.0285264)
Supplement: S1 Table — (DOCX) [file pone.0285264.s003.docx]

**Table S1.** Mean, minimum and maximum wear scores for the Colombian sample investigated (abbreviations as in the main text).

| **Teeth** | **Mean** | **Minimum** | **Maximum** |
| --- | --- | --- | --- |
| UI1 | 1.58 | 1 | 3 |
| UI2 | 1.38 | 1 | 2 |
| UC | 1.84 | 1 | 3 |
| UP3 | 0.98 | 1 | 2 |
| UP4 | 1.42 | 1 | 3 |
| UM1 | 2.32 | 1 | 4 |
| UM2 | 2.22 | 1 | 3 |
| LI1 | 1.64 | 1 | 3 |
| LI2 | 1.42 | 1 | 3 |
| LC | 1.88 | 1 | 3 |
| LP3 | 1.12 | 1 | 3 |
| LP4 | 1.6 | 1 | 3 |
| LM1 | 2.4 | 1 | 4 |
| LM2 | 2.3 | 1 | 4 |
