# Supplementary material for: Dental size variation in admixed Latin Americans: Effects of age, sex and genomic ancestry
Source: PLoS One. 2023 May 4;18(5):e0285264. doi: 10.1371/journal.pone.0285264 (PMC10159210; doi:10.1371/journal.pone.0285264)
Supplement: S2 Table — (DOCX) [file pone.0285264.s004.docx]

**Table S2.** Paired *t*-test results and error percentage validating scanner reproducibility (abbreviations as in the main text).

| **N=15** | **Paired t-test (t-value)** | **Paired t-test (α=0.05; *p*-value)** | **Error percentage** |
| --- | --- | --- | --- |
| UI1 MD | 0.504 | 0.61 | 1.10% |
| UI2 MD | -1.060 | 0.29 | 2.90% |
| UC MD | 0.471 | 0.64 | 1.20% |
| UP3 MD | 0.769 | 0.44 | 1.40% |
| UP4 MD | -0.192 | 0.84 | 1.40% |
