# Supplementary material for: Dental size variation in admixed Latin Americans: Effects of age, sex and genomic ancestry
Source: PLoS One. 2023 May 4;18(5):e0285264. doi: 10.1371/journal.pone.0285264 (PMC10159210; doi:10.1371/journal.pone.0285264)
Supplement: S3 Table — Significant differences are in bold. (DOCX) [file pone.0285264.s005.docx]

**Table S3.** Intraclass correlation coefficients for 28 measurements in the admixed Latin American sample investigated (abbreviations as in the main text). Significant differences are in bold.

| **Teeth** | **Measure** | **ICC** | **P-value** |
| --- | --- | --- | --- |
| UI1 | MD | 0.73916134 | **0.001** |
| UI2 | MD | 0.79387256 | **0.001** |
| UC | MD | 0.72118594 | **0.001** |
| UP3 | MD | 0.91035951 | **0.001** |
| UP4 | MD | 0.86942212 | **0.001** |
| UM1 | MD | 0.79698158 | **0.001** |
| UM2 | MD | 0.85989419 | **0.001** |
| LI1 | MD | 0.71116578 | **0.001** |
| LI2 | MD | 0.87841542 | **0.001** |
| LC | MD | 0.79159208 | **0.001** |
| LP3 | MD | 0.88472851 | **0.001** |
| LP4 | MD | 0.86644861 | **0.001** |
| LM1 | MD | 0.89959622 | **0.001** |
| LM2 | MD | 0.81986649 | **0.001** |
| UI1 | BL | 0.85115456 | **0.001** |
| UI2 | BL | 0.83576806 | **0.001** |
| UC | BL | 0.86973030 | **0.001** |
| UP3 | BL | 0.72462725 | **0.001** |
| UP4 | BL | 0.90468988 | **0.001** |
| UM1 | BL | 0.89710579 | **0.001** |
| UM2 | BL | 0.90636107 | **0.001** |
| LI1 | BL | 0.85523337 | **0.001** |
| LI2 | BL | 0.80678755 | **0.001** |
| LC | BL | 0.88416325 | **0.001** |
| LP3 | BL | 0.90053587 | **0.001** |
| LP4 | BL | 0.85047419 | **0.001** |
| LM1 | BL | 0.85489730 | **0.001** |
| LM2 | BL | 0.83946952 | **0.001** |
