# Supplementary material for: Dental size variation in admixed Latin Americans: Effects of age, sex and genomic ancestry
Source: PLoS One. 2023 May 4;18(5):e0285264. doi: 10.1371/journal.pone.0285264 (PMC10159210; doi:10.1371/journal.pone.0285264)
Supplement: S4 Table — (DOCX) [file pone.0285264.s006.docx]

**Table S4.** Results of the Shapiro-Wilk test evaluating the assumption of normality for 28 MD and BL diameters investigated (abbreviations as in the main text).

| **Tooth** | **Measure** | **Statistic** | **Significance** |
| --- | --- | --- | --- |
| UI1 | MD | 0.997 | 0.451 |
| UI2 | MD | 0.998 | 0.783 |
| UC | MD | 0.996 | 0.607 |
| UP3 | MD | 0.997 | 0.557 |
| UP4 | MD | 0.997 | 0.579 |
| UM1 | MD | 0.996 | 0.099 |
| UM2 | MD | 0.998 | 0.887 |
| LI1 | MD | 0.997 | 0.478 |
| LI2 | MD | 0.998 | 0.630 |
| LC | MD | 0.997 | 0.273 |
| LP3 | MD | 0.997 | 0.569 |
| LP4 | MD | 0.998 | 0.855 |
| LM1 | MD | 0.998 | 0.695 |
| LM2 | MD | 0.996 | 0.356 |
| UI1 | BL | 0.997 | 0.314 |
| UI2 | BL | 0.996 | 0.179 |
| UC | BL | 0.997 | 0.408 |
| UP3 | BL | 0.999 | 0.979 |
| UP4 | BL | 0.996 | 0.185 |
| UM1 | BL | 0.998 | 0.853 |
| UM2 | BL | 0.997 | 0.34 |
| LI1 | BL | 0.996 | 0.188 |
| LI2 | BL | 0.996 | 0.142 |
| LC | BL | 0.996 | 0.144 |
| LP3 | BL | 0.996 | 0.209 |
| LP4 | BL | 0.998 | 0.729 |
| LM1 | BL | 0.996 | 0.254 |
| LM2 | BL | 0.997 | 0.311 |
