# Supplementary material for: Dental size variation in admixed Latin Americans: Effects of age, sex and genomic ancestry
Source: PLoS One. 2023 May 4;18(5):e0285264. doi: 10.1371/journal.pone.0285264 (PMC10159210; doi:10.1371/journal.pone.0285264)
Supplement: S5 Table — (DOCX) [file pone.0285264.s007.docx]

**Table S5.** Descriptive statistics of 28 tooth crown raw measurements for the Colombian sample investigated (abbreviations as in the main text).

| **Tooth** | **Measure** | **Median** | **Min** | **Max** | **SD** | **CV** |
| --- | --- | --- | --- | --- | --- | --- |
| UI1 | MD | 8.804 | 7.408 | 10.335 | 0.530 | 6.024 |
| UI2 | MD | 7.079 | 5.314 | 8.687 | 0.574 | 8.136 |
| UC | MD | 8.112 | 6.743 | 9.481 | 0.500 | 6.179 |
| UP3 | MD | 7.300 | 6.120 | 8.498 | 0.419 | 5.739 |
| UP4 | MD | 7.005 | 5.790 | 8.380 | 0.426 | 6.077 |
| UM1 | MD | 10.607 | 9.236 | 12.179 | 0.547 | 5.149 |
| UM2 | MD | 10.104 | 8.117 | 12.157 | 0.660 | 6.517 |
| LI1 | MD | 5.484 | 4.444 | 6.478 | 0.358 | 6.528 |
| LI2 | MD | 6.129 | 4.846 | 7.296 | 0.401 | 6.546 |
| LC | MD | 6.972 | 5.729 | 8.406 | 0.472 | 6.762 |
| LP3 | MD | 7.263 | 6.081 | 8.534 | 0.415 | 5.698 |
| LP4 | MD | 7.332 | 5.915 | 8.596 | 0.439 | 5.978 |
| LM1 | MD | 11.104 | 9.467 | 12.701 | 0.553 | 4.973 |
| LM2 | MD | 10.599 | 8.969 | 12.477 | 0.636 | 6.001 |
| UI1 | BL | 7.212 | 5.920 | 8.598 | 0.493 | 6.840 |
| UI2 | BL | 6.386 | 4.968 | 7.894 | 0.531 | 8.316 |
| UC | BL | 8.079 | 6.612 | 9.738 | 0.557 | 6.873 |
| UP3 | BL | 9.315 | 7.699 | 10.834 | 0.502 | 5.395 |
| UP4 | BL | 9.283 | 7.662 | 10.858 | 0.550 | 5.895 |
| UM1 | BL | 10.781 | 9.159 | 12.630 | 0.578 | 5.353 |
| UM2 | BL | 10.904 | 9.078 | 12.979 | 0.688 | 6.302 |
| LI1 | BL | 6.008 | 4.756 | 7.072 | 0.426 | 7.061 |
| LI2 | BL | 6.303 | 5.258 | 7.365 | 0.394 | 6.235 |
| LC | BL | 7.445 | 5.987 | 9.139 | 0.542 | 7.268 |
| LP1 | BL | 7.719 | 6.277 | 9.220 | 0.480 | 6.209 |
| LP2 | BL | 8.263 | 6.829 | 9.720 | 0.511 | 6.183 |
| LM1 | BL | 10.174 | 8.754 | 11.998 | 0.514 | 5.047 |
| LM2 | BL | 9.927 | 8.481 | 12.028 | 0.574 | 5.766 |
