# Supplementary material for: Dental size variation in admixed Latin Americans: Effects of age, sex and genomic ancestry
Source: PLoS One. 2023 May 4;18(5):e0285264. doi: 10.1371/journal.pone.0285264 (PMC10159210; doi:10.1371/journal.pone.0285264)
Supplement: S6 Table — (DOCX) [file pone.0285264.s008.docx]

**Table S6.** Descriptive statistics of 3 dental indexes for the Colombian sample investigated (abbreviations as in the main text).

| **Tooth** | **Index** | **Median** | **Min** | **Max** | **SD** | **CV** |
| --- | --- | --- | --- | --- | --- | --- |
| UI1 | CM | 8.020 | 6.902 | 9.198 | 0.426 | 5.326 |
| UI2 | CM | 6.727 | 5.459 | 8.132 | 0.455 | 6.775 |
| UC | CM | 8.101 | 6.830 | 9.487 | 0.459 | 5.668 |
| UP3 | CM | 8.322 | 6.978 | 9.563 | 0.418 | 5.036 |
| UP4 | CM | 8.158 | 6.726 | 9.490 | 0.435 | 5.328 |
| UM1 | CM | 10.711 | 9.372 | 12.186 | 0.49 | 4.578 |
| UM2 | CM | 10.508 | 8.895 | 12.164 | 0.586 | 5.576 |
| LI1 | CM | 5.743 | 4.813 | 6.645 | 0.318 | 5.522 |
| LI2 | CM | 6.229 | 5.362 | 7.171 | 0.317 | 5.089 |
| LC | CM | 7.227 | 5.987 | 8.608 | 0.443 | 6.132 |
| LP3 | CM | 7.515 | 6.295 | 8.682 | 0.387 | 5.158 |
| LP4 | CM | 7.812 | 6.461 | 8.988 | 0.417 | 5.339 |
| LM1 | CM | 10.639 | 9.204 | 12.321 | 0.473 | 4.440 |
| LM2 | CM | 10.270 | 8.886 | 11.834 | 0.541 | 5.260 |
| UI1 | CI | 0.819 | 0.655 | 1.022 | 0.059 | 7.154 |
| UI2 | CI | 0.905 | 0.692 | 1.186 | 0.086 | 9.406 |
| UC | CI | 1.000 | 0.803 | 1.274 | 0.066 | 6.559 |
| UP3 | CI | 1.275 | 1.090 | 1.515 | 0.06 | 4.692 |
| UP4 | CI | 1.333 | 1.098 | 1.586 | 0.072 | 5.416 |
| UM1 | CI | 1.019 | 0.865 | 1.232 | 0.052 | 5.143 |
| UM2 | CI | 1.078 | 0.915 | 1.398 | 0.069 | 6.371 |
| LI1 | CI | 1.103 | 0.825 | 1.382 | 0.089 | 8.047 |
| LI2 | CI | 1.030 | 0.829 | 1.320 | 0.080 | 7.781 |
| LC | CI | 1.065 | 0.886 | 1.360 | 0.074 | 6.924 |
| LP3 | CI | 1.061 | 0.867 | 1.280 | 0.064 | 6.014 |
| LP4 | CI | 1.130 | 0.799 | 1.321 | 0.065 | 5.809 |
| LM1 | CI | 0.916 | 0.810 | 1.058 | 0.043 | 4.654 |
| LM2 | CI | 0.940 | 0.806 | 1.176 | 0.050 | 5.290 |
| UI1 | CA | 63.600 | 46.841 | 84.251 | 6.794 | 10.689 |
| UI2 | CA | 45.049 | 29.754 | 66.067 | 6.098 | 13.510 |
| UC | CA | 65.599 | 46.637 | 89.990 | 7.471 | 11.350 |
| UP3 | CA | 68.019 | 48.174 | 89.880 | 6.844 | 10.070 |
| UP4 | CA | 65.184 | 44.365 | 88.190 | 6.966 | 10.638 |
| UM1 | CA | 114.677 | 87.838 | 148.503 | 10.486 | 9.130 |
| UM2 | CA | 110.300 | 78.932 | 147.549 | 12.337 | 11.147 |
| LI1 | CA | 32.932 | 23.161 | 44.038 | 3.639 | 10.992 |
| LI2 | CA | 38.748 | 28.753 | 51.401 | 3.941 | 10.165 |
| LC | CA | 52.041 | 35.847 | 74.029 | 6.414 | 12.276 |
| LP3 | CA | 56.328 | 39.621 | 75.082 | 5.817 | 10.301 |
| LP4 | CA | 60.843 | 41.441 | 80.384 | 6.476 | 10.653 |
| LM1 | CA | 112.834 | 84.628 | 151.670 | 10.091 | 8.900 |
| LM2 | CA | 105.202 | 78.949 | 139.956 | 11.126 | 10.516 |
