# Supplementary material for: Dental size variation in admixed Latin Americans: Effects of age, sex and genomic ancestry
Source: PLoS One. 2023 May 4;18(5):e0285264. doi: 10.1371/journal.pone.0285264 (PMC10159210; doi:10.1371/journal.pone.0285264)
Supplement: S7 Table — (DOCX) [file pone.0285264.s009.docx]

**Table S7.** Descriptive statistics of 28 tooth crown measurements for the Native American sample investigated (abbreviations as in the main text).

| **Tooth** | **Measure** | **Median** | **Min** | **Max** | **SD** | **CV** |
| --- | --- | --- | --- | --- | --- | --- |
| UI1 | MD | 8.882 | 7.650 | 9.960 | 0.394 | 4.425 |
| UI2 | MD | 7.501 | 5.750 | 8.830 | 0.494 | 6.590 |
| UC | MD | 8.220 | 7.100 | 9.200 | 0.388 | 4.720 |
| UP3 | MD | 7.450 | 6.430 | 8.490 | 0.390 | 5.237 |
| UP4 | MD | 7.150 | 5.880 | 8.360 | 0.412 | 5.748 |
| UM1 | MD | 11.020 | 9.450 | 12.360 | 0.520 | 4.712 |
| UM2 | MD | 10.510 | 8.520 | 11.850 | 0.592 | 5.663 |
| LI1 | MD | 5.567 | 4.850 | 6.430 | 0.277 | 4.973 |
| LI2 | MD | 6.380 | 5.420 | 7.420 | 0.371 | 5.802 |
| LC | MD | 7.250 | 5.770 | 8.430 | 0.410 | 5.641 |
| LP3 | MD | 7.170 | 5.970 | 8.140 | 0.395 | 5.492 |
| LP4 | MD | 7.390 | 6.100 | 8.680 | 0.457 | 6.165 |
| LM1 | MD | 11.830 | 10.270 | 13.150 | 0.522 | 4.416 |
| LM2 | MD | 11.420 | 9.290 | 13.000 | 0.667 | 5.852 |
| UI1 | BL | 7.430 | 6.110 | 8.570 | 0.406 | 5.465 |
| UI2 | BL | 6.702 | 5.040 | 7.970 | 0.424 | 6.320 |
| UC | BL | 8.380 | 7.150 | 9.680 | 0.497 | 5.909 |
| UP3 | BL | 9.580 | 8.060 | 11.030 | 0.522 | 5.430 |
| UP4 | BL | 9.420 | 7.850 | 10.850 | 0.534 | 5.687 |
| UM1 | BL | 11.880 | 10.450 | 13.150 | 0.516 | 4.352 |
| UM2 | BL | 11.700 | 10.050 | 13.250 | 0.631 | 5.385 |
| LI1 | BL | 5.830 | 4.780 | 6.970 | 0.343 | 5.897 |
| LI2 | BL | 6.223 | 5.260 | 7.290 | 0.352 | 5.668 |
| LC | BL | 7.700 | 6.320 | 9.080 | 0.515 | 6.691 |
| LP3 | BL | 7.980 | 6.730 | 9.310 | 0.487 | 6.095 |
| LP4 | BL | 8.370 | 6.950 | 9.810 | 0.479 | 5.722 |
| LM1 | BL | 11.090 | 9.820 | 12.340 | 0.491 | 4.433 |
| LM2 | BL | 10.670 | 9.080 | 12.020 | 0.542 | 5.098 |
