# Supplementary material for: Dental size variation in admixed Latin Americans: Effects of age, sex and genomic ancestry
Source: PLoS One. 2023 May 4;18(5):e0285264. doi: 10.1371/journal.pone.0285264 (PMC10159210; doi:10.1371/journal.pone.0285264)
Supplement: S8 Table — (DOCX) [file pone.0285264.s010.docx]

**Table S8.** Descriptive statistics of 28 tooth crown measurements for the European sample investigated (abbreviations as in the main text).

| **Tooth** | **Measure** | **Median** | **Min** | **Max** | **SD** | **CV** |
| --- | --- | --- | --- | --- | --- | --- |
| UI1 | MD | 8.390 | 7.260 | 10.010 | 0.446 | 5.300 |
| UI2 | MD | 6.580 | 5.340 | 8.030 | 0.500 | 7.591 |
| UC | MD | 7.512 | 6.570 | 8.870 | 0.398 | 5.267 |
| UP3 | MD | 6.700 | 5.750 | 8.300 | 0.393 | 5.854 |
| UP4 | MD | 6.510 | 5.590 | 8.130 | 0.385 | 5.891 |
| UM1 | MD | 10.460 | 9.110 | 12.120 | 0.520 | 4.959 |
| UM2 | MD | 9.710 | 8.140 | 11.310 | 0.542 | 5.573 |
| LI1 | MD | 5.210 | 4.590 | 5.940 | 0.252 | 4.825 |
| LI2 | MD | 5.800 | 5.010 | 6.750 | 0.337 | 5.786 |
| LC | MD | 6.660 | 5.590 | 7.900 | 0.396 | 5.951 |
| LP3 | MD | 6.705 | 5.910 | 7.990 | 0.388 | 5.762 |
| LP4 | MD | 6.940 | 6.000 | 8.400 | 0.420 | 6.052 |
| LM1 | MD | 11.070 | 9.680 | 12.420 | 0.519 | 4.690 |
| LM2 | MD | 10.620 | 9.120 | 12.790 | 0.607 | 5.698 |
| UI1 | BL | 7.150 | 6.000 | 8.500 | 0.390 | 5.444 |
| UI2 | BL | 6.320 | 4.940 | 7.950 | 0.439 | 6.907 |
| UC | BL | 8.230 | 6.840 | 9.680 | 0.530 | 6.433 |
| UP3 | BL | 8.850 | 7.660 | 10.580 | 0.550 | 6.202 |
| UP4 | BL | 9.020 | 7.700 | 10.910 | 0.532 | 5.861 |
| UM1 | BL | 11.360 | 10.140 | 12.830 | 0.550 | 4.838 |
| UM2 | BL | 11.400 | 9.240 | 13.440 | 0.692 | 6.056 |
| LI1 | BL | 5.797 | 4.790 | 6.830 | 0.340 | 5.863 |
| LI2 | BL | 6.150 | 5.200 | 7.190 | 0.359 | 5.815 |
| LC | BL | 7.590 | 6.220 | 9.200 | 0.578 | 7.621 |
| LP3 | BL | 7.620 | 6.520 | 9.110 | 0.492 | 6.449 |
| LP4 | BL | 8.180 | 6.900 | 9.530 | 0.505 | 6.148 |
| LM1 | BL | 10.447 | 9.100 | 11.760 | 0.481 | 4.614 |
| LM2 | BL | 10.030 | 8.500 | 11.750 | 0.565 | 5.617 |
