# Supplementary material for: Dental size variation in admixed Latin Americans: Effects of age, sex and genomic ancestry
Source: PLoS One. 2023 May 4;18(5):e0285264. doi: 10.1371/journal.pone.0285264 (PMC10159210; doi:10.1371/journal.pone.0285264)
Supplement: S9 Table — (DOCX) [file pone.0285264.s011.docx]

**Table S9.** Descriptive statistics of 28 tooth crown measurements for the African sample investigated (abbreviations as in the main text).

| **Tooth** | **Measure** | **Median** | **Min** | **Max** | **SD** | **CV** |
| --- | --- | --- | --- | --- | --- | --- |
| UI1 | MD | 8.873 | 7.680 | 10.330 | 0.364 | 4.097 |
| UI2 | MD | 7.177 | 5.940 | 8.570 | 0.428 | 5.968 |
| UC | MD | 7.950 | 6.720 | 9.220 | 0.415 | 5.210 |
| UP3 | MD | 7.445 | 6.480 | 8.750 | 0.415 | 5.569 |
| UP4 | MD | 7.055 | 6.000 | 8.040 | 0.439 | 6.231 |
| UM1 | MD | 10.985 | 9.640 | 12.330 | 0.545 | 4.958 |
| UM2 | MD | 10.440 | 8.580 | 11.960 | 0.671 | 6.415 |
| LI1 | MD | 5.526 | 4.590 | 6.084 | 0.254 | 4.614 |
| LI2 | MD | 6.188 | 5.340 | 7.000 | 0.335 | 5.424 |
| LC | MD | 7.179 | 6.330 | 8.260 | 0.425 | 5.906 |
| LP3 | MD | 7.400 | 6.330 | 8.230 | 0.395 | 5.352 |
| LP4 | MD | 7.495 | 6.010 | 8.630 | 0.446 | 5.987 |
| LM1 | MD | 11.751 | 10.370 | 13.080 | 0.497 | 4.250 |
| LM2 | MD | 11.190 | 9.180 | 12.920 | 0.698 | 6.247 |
| UI1 | BL | 7.449 | 6.360 | 8.510 | 0.354 | 4.771 |
| UI2 | BL | 6.763 | 5.500 | 8.000 | 0.396 | 5.871 |
| UC | BL | 8.580 | 7.120 | 9.950 | 0.525 | 6.123 |
| UP3 | BL | 9.670 | 8.190 | 10.740 | 0.518 | 5.351 |
| UP4 | BL | 9.595 | 8.560 | 10.980 | 0.499 | 5.199 |
| UM1 | BL | 11.660 | 10.470 | 12.940 | 0.518 | 4.453 |
| UM2 | BL | 11.890 | 10.120 | 13.510 | 0.647 | 5.455 |
| LI1 | BL | 5.913 | 4.940 | 6.580 | 0.304 | 5.173 |
| LI2 | BL | 6.311 | 5.220 | 7.020 | 0.310 | 4.935 |
| LC | BL | 7.882 | 6.210 | 9.180 | 0.502 | 6.400 |
| LP3 | BL | 8.300 | 7.130 | 9.420 | 0.489 | 5.878 |
| LP4 | BL | 8.610 | 7.330 | 9.820 | 0.494 | 5.750 |
| LM1 | BL | 10.740 | 9.710 | 12.320 | 0.485 | 4.496 |
| LM2 | BL | 10.480 | 9.050 | 12.040 | 0.595 | 5.672 |
